# Large language models for complex decision-making in colorectal cancer with peritoneal metastases

**DOI:** 10.3389/fmed.2026.1997728

**Published:** 2026-10-28

**Authors:** Zhongwei Qi, Dongdong Wang, Shuxia Liang, Yamei Li, Muju Yue, Yang Shi, Zhendong Liu

**Affiliations:** 1 College of Nursing, Gansu University of Chinese Medicine, Lanzhou, Gansu, China; 2 Department of Emergency Medicine, The 940th Hospital of Joint Logistics Support force of Chinese People's Liberation Army, Lanzhou, Gansu, China

**Keywords:** clinical decision-making, colorectal cancer, cytoreductive surgery, expert consensus, large language models, peritoneal metastases

## Abstract

**Background:**

Large language models (LLMs) are increasingly evaluated for oncology decision support, but their performance in colorectal cancer with peritoneal metastases (CRC-PM) remains uncertain. We benchmarked 10 contemporary LLMs against an expert-consensus framework.

**Methods:**

Forty-eight synthetic vignettes were derived from a 2026 CRC-PM consensus guideline, including 36 primary scenarios and 12 counterfactual scenarios forming six pairs. Each model completed every vignette three times using a frozen zero-shot structured prompt. The primary endpoint was prompt-aligned structured Full concordance; contradiction-only and full-semantic adjudication were sensitivity analyses. Two blinded attending colorectal surgeons, each with 5 years of clinical experience, independently validated the reference classifications.

**Results:**

Among 1,080 primary responses, structured scoring classified 609 (56.4%) as Full, 117 (10.8%) as Partial, and 354 (32.8%) as Discordant. Model-specific structured Full concordance ranged from 49.1% to 63.0%; neither the Friedman test (*P* = .104) nor clustered GEE (*P* = .186) supported a robust model effect. Contradiction-only scoring was similar (56.2% Full), whereas full-semantic adjudication reduced Full concordance to 45.5%. Excluding prompt-development scenarios lowered absolute concordance but did not change the inferential conclusion. Expert agreement was 93.8%–97.9% across decision domains (Cohen kappa 0.898–0.948). Counterfactual and metachronous risk-management analyses showed wide uncertainty, and 21 clinically consequential discordance flags arose from five scenarios.

**Conclusions:**

LLM performance in CRC-PM was sensitive to scoring definition and did not establish a stable between-model hierarchy. Prospective clinician-plus-LLM studies are required before clinical benefit can be inferred.

## 1 Introduction

Peritoneal metastases from colorectal cancer occur synchronously or metachronously in an estimated 15% to 40% of patients over the course of disease and historically have been associated with some of the poorest outcomes among metastatic sites (1). Over the past two decades, management of colorectal cancer with peritoneal metastases (CRC-PM) has shifted from a uniformly palliative paradigm toward a multimodal approach in which carefully selected patients may achieve prolonged survival through cytoreductive surgery (CRS), intraperitoneal chemotherapy (IPCT), and systemic therapy delivered in coordinated sequence (2). Yet the evidence supporting individual treatment components and their timing remains unsettled. PRODIGE 7 found no overall-survival benefit from adding short-duration oxaliplatin hyperthermic intraperitoneal chemotherapy (HIPEC) to otherwise complete cytoreduction, CAIRO6 found no significant benefit of perioperative systemic therapy over surgery alone for resectable peritoneal-only disease, and COLOPEC found no reduction in peritoneal recurrence with adjuvant HIPEC after resection of high-risk colon cancer (3–5). These uncertainties are compounded by marked clinical heterogeneity: prognosis and treatment tolerance vary with histology, timing of peritoneal dissemination, performance status, disease burden, and the likelihood of achieving complete cytoreduction. Signet-ring morphology, for example, is associated with particularly adverse outcomes and a greater risk of non-therapeutic laparotomy (6). Consequently, no single treatment algorithm applies uniformly across patients.

Because no single randomized trial or biomarker adjudicates most of these decisions, contemporary CRC-PM management relies heavily on structured expert-consensus processes to translate heterogeneous, often lower-level evidence into clinically actionable pathways (7). Modified Delphi methodology, in which experts iteratively rate and refine recommendations across successive rounds until a prespecified agreement threshold is reached, has become an established approach to guideline development and multidisciplinary oncology governance (8). The recently updated national consensus guideline for CRC-PM applied this approach to construct synchronous and metachronous management pathways spanning referral, initial systemic therapy, restaging, CRS and IPCT candidacy, metachronous risk stratification, recurrence, and surveillance (9). This framework provides a structured, block-based expert-consensus foundation from which clinically actionable reference decisions can be operationalized for benchmarking. Importantly, Delphi agreement was assessed at the pathway-block level rather than for each atomic recommendation, so block-level agreement is best interpreted as contextual consensus strength rather than item-specific support.

In parallel, large language models (LLMs) have progressed rapidly in their capacity to encode and apply clinical knowledge, moving from strong performance on medical question-answering benchmarks toward increasingly complex decision-support tasks (10). Oncology applications have expanded accordingly, from broad benchmarking frameworks to direct comparisons between LLM-generated recommendations and multidisciplinary tumor board decisions (11, 12). Reported concordance has varied across tumor types and clinical domains, including breast cancer, neuro-oncology radiotherapy, lung and thoracic malignancies, stage II colon cancer, and guideline-based rectal cancer decision-making (13–19). Prospective work has also explored locally deployed models as adjuncts to real-world head and neck tumor board discussions (20). More specifically, a recent CRC-PM study compared GPT-5 with two surgical oncologists across 15 hypothetical CRS + HIPEC eligibility scenarios and reported 86.7% agreement with expert assessments (21). However, that study evaluated a single model at a narrowly defined eligibility decision point and did not examine longitudinal management pathways, repeated-run stability, uncertainty recognition, or counterfactual responsiveness to changes in clinically decisive variables.

Most existing oncology benchmarks therefore leave several questions unresolved that are especially important in a decision-dense disease such as CRC-PM. They commonly evaluate isolated decisions rather than linked management steps extending from referral and systemic therapy through restaging, surgical selection, recurrence, and surveillance; they rarely distinguish recognition of a clinically relevant risk feature from correct translation of that feature into management; and few directly test whether a model changes its recommendation in the appropriate direction when only one decisive feature is altered. Self-reported certainty is also an imperfect signal of reliability, because LLMs can remain highly confident when answers are wrong and the relationship between expressed confidence and accuracy varies across models and specialties (22–24).

To address these gaps, we benchmarked 10 contemporary LLMs against an expert-consensus framework for CRC-PM (9). The study evaluated prompt-aligned structured concordance, stricter semantic sensitivity analyses, repeated-run consistency, translation of metachronous risk recognition into management, directional robustness under six prespecified counterfactual perturbations, the relationship between self-reported certainty and decision correctness, and a prespecified taxonomy of clinically consequential discordance patterns.

## 2 Methods

### 2.1 Study design and expert-consensus reference standard

This benchmark study evaluated the clinical decision-making performance of 10 LLMs in CRC-PM. The reference framework was derived from the updated consensus guideline reported by Schultz et al., which used a modified Delphi process to construct synchronous and metachronous management pathways (9). Because Delphi voting was conducted at the pathway-block level rather than for individual atomic recommendations, round-2 agreement percentages were treated as contextual measures of consensus strength and were not interpreted as item-level probabilities of correctness. The overall study design and analytic workflow are summarized in Figure 1.

**Figure 1:**
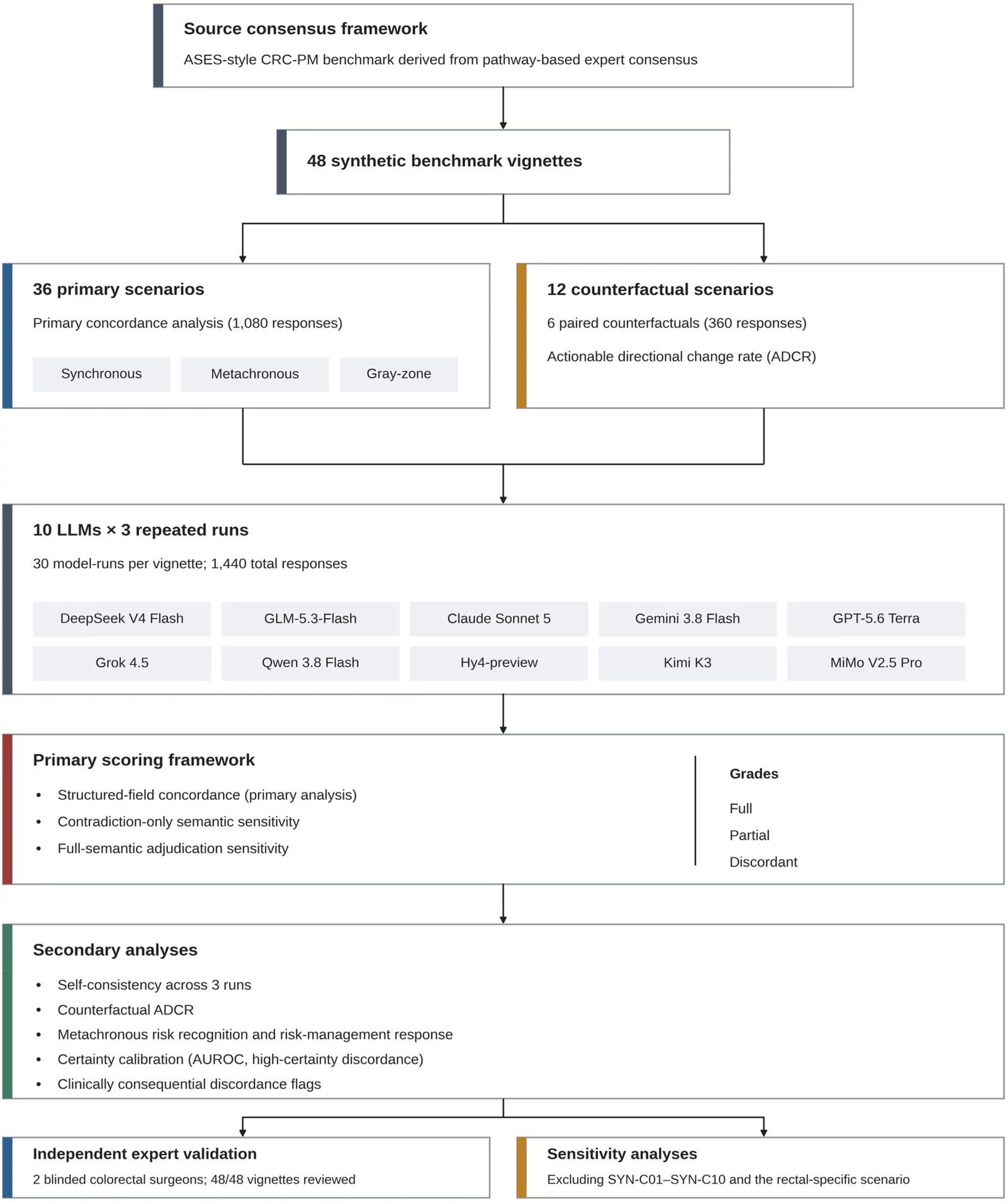
Study design and workflow. Forty-eight synthetic vignettes (36 primary scenarios and 12 counterfactual scenarios forming six pairs), derived from the pathway-based consensus framework, were each answered three times by 10 LLMs under a frozen zero-shot prompt (1,440 responses). Primary scoring used structured-field concordance (Full, Partial, or Discordant), with contradiction-only and full-semantic adjudication as sensitivity analyses. Two blinded colorectal surgeons independently reviewed all 48 vignettes; sensitivity analyses excluded the prompt-development overlap (SYN-C01 to SYN-C10) and the rectal-specific scenario. ADCR, actionable directional change rate; AUROC, area under the receiver operating characteristic curve; CRC-PM, colorectal cancer with peritoneal metastases; LLM, large language model.

The consensus pathways were decomposed into actionable decisions spanning referral and multidisciplinary review, initial systemic therapy, restaging, CRS candidacy and timing, IPCT positioning, metachronous risk stratification, repeat CRS, nonoperative management, and surveillance. CRS and IPCT were scored separately so that a model could appropriately support CRS while maintaining a cautious or uncertain position on IPCT.

### 2.2 Benchmark construction and clinical definitions

The final benchmark contained 48 synthetic clinical vignettes: 28 core cases, 8 gray-zone cases, and 12 counterfactual cases. The 12 counterfactual cases formed six strict pairs in which a single clinically decisive feature was changed: performance status, predicted completeness of cytoreduction, disease progression after first-line systemic therapy, number of liver metastases, histology (conventional vs. signet-ring), or primary-tumor sidedness. Thirty-six non-counterfactual cases (23 synchronous and 13 metachronous) comprised the primary concordance analysis; the counterfactual cases were analyzed separately.

For benchmark construction, synchronous peritoneal metastases were operationally defined as peritoneal disease present at the initial colorectal cancer presentation, whereas metachronous peritoneal metastases referred to disease detected after previous resection of the primary colorectal tumor. Within the metachronous pathway, a disease-free interval of less than 12 months was treated as a high-risk feature in accordance with the source consensus framework. Where treatment duration was relevant to the tested node, the reference rule operationalized an approximately six-month systemic-treatment strategy as either 3–6 months before reassessment or completion of approximately six months total when preoperative treatment had already been given.

Each vignette was mapped to a reference primary action and, when relevant, a CRS position, IPCT position, and metachronous risk category. Only fields relevant to the decision node were scored. The vignettes were designed as decision-isolation scenarios rather than complete patient records. Clinical attributes were included only when relevant to the tested node; unstated findings were not inferred by the investigators or supplied to the models. Supplementary Table S1 reports, for every vignette, the availability of age, sex, ECOG performance status, T and N category, PCI, extraperitoneal disease, histology or grade, molecular status, sidedness, method of peritoneal-metastasis ascertainment, and systemic-therapy regimen, line, duration, and response.

### 2.3 Prompt development and separation from formal evaluation

Prompt development proceeded through four iterative stages. First, the source pathways were decomposed into decision nodes. Second, an initial structured prompt was developed to elicit a single next action together with separate CRS and IPCT positions, self-reported certainty, and a brief rationale; the metachronous-risk field and explicit label definitions were subsequently added. Third, the benchmark was expanded to include core, gray-zone, and counterfactual scenarios, and a 10-case DeepSeek pilot using SYN-C01 through SYN-C10 was conducted. Fourth, pilot-informed revisions were incorporated into Benchmark V2 and the final V3 prompt, including acceptance of clinically equivalent action labels, scoring only fields relevant to the decision node, clarification of the temporal interpretation of CRS and IPCT positions, and case-specific semantic-review rules. Revisions targeted ambiguities exposed by pilot responses rather than optimization of a model ranking. The vignette set, reference actions, scoring rules, and V3 prompt were frozen before formal multi-model testing began on September 8, 2026.

Because SYN-C01 through SYN-C10 were used during prompt development and remained in the formal benchmark, development and test sets were not completely disjoint. A sensitivity analysis therefore excluded all 10 overlapping scenarios, reducing the primary analysis from 36 to 26 independent scenarios.

### 2.4 Models, prompting, and inference protocol

The evaluated model aliases were DeepSeek V4 Flash, GLM-5.3-Flash, Claude Sonnet 5, Gemini 3.8 Flash, GPT-5.6 Terra, Grok 4.5, Qwen 3.8 Flash, Hy4-preview, Kimi K3, and MiMo V2.5 Pro. They were accessed through provider endpoints used in the study. Complete access routes and settings are reported in Supplementary Table S2.

A single frozen zero-shot prompt was used across models. No few-shot examples, chain-of-thought request, role-playing persona, retrieval augmentation, web search, or external clinical tools were used. Required fields were primary_action, crs_position, ipct_position, metachronous_risk, certainty (integer 0–100), and a rationale of no more than 50 words. Structured JSON-object output was requested, but a common strict JSON Schema could not be enforced uniformly across providers. Reasoning or thinking was disabled whenever the interface permitted it; when reasoning could not be disabled, the lowest available reasoning setting was used. Temperature was set to 0 when supported and otherwise omitted. Maximum output length was 1,000 tokens for most models and 4,096 tokens for models requiring additional reasoning-token capacity.

Each of the 48 cases was queried three times per model, yielding 144 responses per model and 1,440 responses overall. Case order was independently randomized within each run using a fixed request-order seed. Testing was performed on September 8–9, 2026. A response was considered technically valid only if the API call succeeded, JSON could be parsed, all required fields were present, and categorical outputs belonged to the allowed label set. Fourteen initial responses with schema or parsing noncompliance were reissued under identical experimental settings and replaced before analysis.

### 2.5 Independent expert validation of the reference standard

The benchmark reference classifications were independently reviewed by two attending colorectal surgeons, each with 5 years of clinical experience (Zijie Yang and Min Zou). Both reviewers independently assessed all 48 vignettes and the corresponding classifications for primary management action, CRS position, IPCT position, and metachronous risk category while blinded to model identity and all model-generated responses. Raw percentage agreement and Cohen kappa were calculated for each domain, and exact four-domain agreement was calculated at the vignette level. Disagreements were adjudicated after independent review. Because adjudication retained the original reference classification for every vignette, the frozen model outputs did not require rerunning or relabeling against a changed reference set.

### 2.6 Outcome definitions and semantic sensitivity analyses

The primary outcome was Full concordance under prompt-aligned structured-field scoring among the 36 non-counterfactual scenarios. A response was classified as Full when all scored structured fields were fully concordant with their prespecified acceptable values; Partial when at least one field was conditionally acceptable without a directly discordant field; and Discordant when at least one scored field was incompatible with the reference decision.

Two semantic sensitivity analyses were performed. In the contradiction-only analysis, a structured grade could be downgraded only when the rationale contained an explicit clinically meaningful contradiction, an incorrect assertion of standard care, or a categorical overstatement of a conditional recommendation. Mere omission of additional surveillance, treatment-duration, reassessment, or radiotherapy details did not trigger downgrading. A stricter full-semantic analysis applied case-specific semantic rules, including omission-based criteria. Semantic review could downgrade but not upgrade the structured grade.

Secondary outcomes included exact four-field self-consistency across the three repeated runs, counterfactual actionable directional change rate (ADCR), metachronous risk recognition rate (RRR), and risk-management response rate (RMR). Exact self-consistency required identical primary_action, crs_position, ipct_position, and metachronous_risk across all three runs of a case. Run-level ADCR described the proportion of the 18 pair-run evaluations per model showing the prespecified direction of change; because the independent unit was the six counterfactual pairs, inferential precision was summarized using pair-level majority success, defined as directionally correct in at least two of three runs. RRR quantified correct LOW/HIGH classification across nine risk-scored metachronous scenarios; RMR quantified translation of high-risk status into an acceptable systemic/next-line management action across eight high-risk scenarios.

Self-reported certainty was analyzed as a 0–100 confidence signal, not as a calibrated probability. High-certainty discordance was defined as a full-semantic Discordant response with certainty of at least 90. Certainty discrimination of Full vs. non-Full responses was summarized using AUROC. Prespecified clinically consequential discordance flags were retained for incorrect treatment sequence, undertreatment, overtreatment, failure to recognize unresectability, inappropriate IPCT, and categorical overstatement of a conditional recommendation. These flags were descriptive benchmark events, not patient-level safety outcomes.

### 2.7 Statistical analysis

The 36 primary scenarios, rather than the 108 repeated responses per model, were treated as the independent clinical units. The scenario count was determined by coverage of distinct consensus decision nodes rather than by a formal power calculation. With 36 independent scenarios, a proportion near 0.50 has a nominal 95% Wilson interval half-width of approximately 15.5 percentage points, so model-specific estimates were expected to be imprecise and the study was interpreted as exploratory.

For structured, contradiction-only, and full-semantic Full concordance, 95% confidence intervals were obtained by nonparametric scenario-cluster bootstrap resampling, preserving all three runs within each resampled scenario (10,000 resamples). Overall model comparisons used a Friedman test applied to scenario-level mean Full-concordance proportions. Pairwise two-sided Wilcoxon signed-rank tests used the Pratt treatment of zero differences and Holm multiplicity adjustment. Population-averaged logistic generalized estimating equations (GEE) with model and run as fixed effects, scenario as the clustering variable, and an exchangeable working correlation structure were used as clustered sensitivity analyses.

Wilson intervals were used for exact self-consistency (48 scenarios per model) and pair-level majority ADCR (six pairs per model). RRR and RMR intervals used scenario-cluster bootstrap resampling of nine and eight independent scenarios, respectively. AUROC confidence intervals used scenario-level bootstrap resampling (2,000 iterations). Block-level associations between Delphi agreement and model certainty or concordance were evaluated with Spearman correlation. All tests were two-sided with *P* < .05 considered statistically significant. Analyses used Python with pandas, SciPy, statsmodels, and scikit-learn.

## 3 Results

### 3.1 Benchmark characteristics and response completeness

Among the 36 primary scenarios, 33 were generic CRC-PM scenarios without colon-vs.-rectal specification, two explicitly specified colonic sidedness, and one (GRY-08) was rectal-specific. Meaningful colon-vs.-rectal stratification was therefore not possible. No primary vignette specified age, sex, T category, molecular status, or a regimen-level systemic-therapy protocol, and the modality used to establish peritoneal metastases was not standardized. These unstated characteristics are reported as “Not specified” in Supplementary Table S1 rather than retrospectively imputed.

The final dataset contained 1,440 schema-valid responses (10 models×48 cases×3 runs), including 1,080 responses from the 36 primary cases and 360 responses from the 12 counterfactual cases. Fourteen initial responses (1.0%) required reissue because of schema or parsing noncompliance: 10 from GLM-5.3-Flash, three from Claude Sonnet 5, and one from Kimi K3. All replacement responses passed the prespecified validation checks.

### 3.2 Independent expert validation

Independent clinical validation demonstrated high reproducibility of the benchmark reference classifications. The two colorectal surgeons agreed on primary management action in 46 of 48 vignettes (95.8%), CRS positioning in 46 (95.8%), IPCT positioning in 45 (93.8%), and metachronous risk classification in 47 (97.9%). Corresponding Cohen kappa coefficients were 0.948, 0.935, 0.898, and 0.944. Exact agreement across all four domains was observed in 43 of 48 vignettes (89.6%). Complete four-domain agreement with the original reference was 93.8% for Expert 1 and 89.6% for Expert 2. Inter-reviewer disagreements occurred in five vignettes; following adjudication, the original benchmark reference classification was retained for all 48 scenarios.

### 3.3 Primary structured concordance and semantic sensitivity analyses

Under the primary structured-field analysis, 609 of 1,080 responses (56.4%) were Full, 117 (10.8%) Partial, and 354 (32.8%) Discordant. Model-specific Full concordance ranged from 49.1% to 63.0%, with wide and substantially overlapping scenario-clustered confidence intervals (Figure 2 and Table 1). The scenario-level Friedman test did not show significant overall heterogeneity (chi-square = 14.56, df = 9, *P* = .104), the minimum Holm-adjusted pairwise *P* value was.649, and the scenario-clustered GEE model effect was also non-significant (Wald chi-square = 12.50, df = 9, *P* = .186).

**Figure 2:**
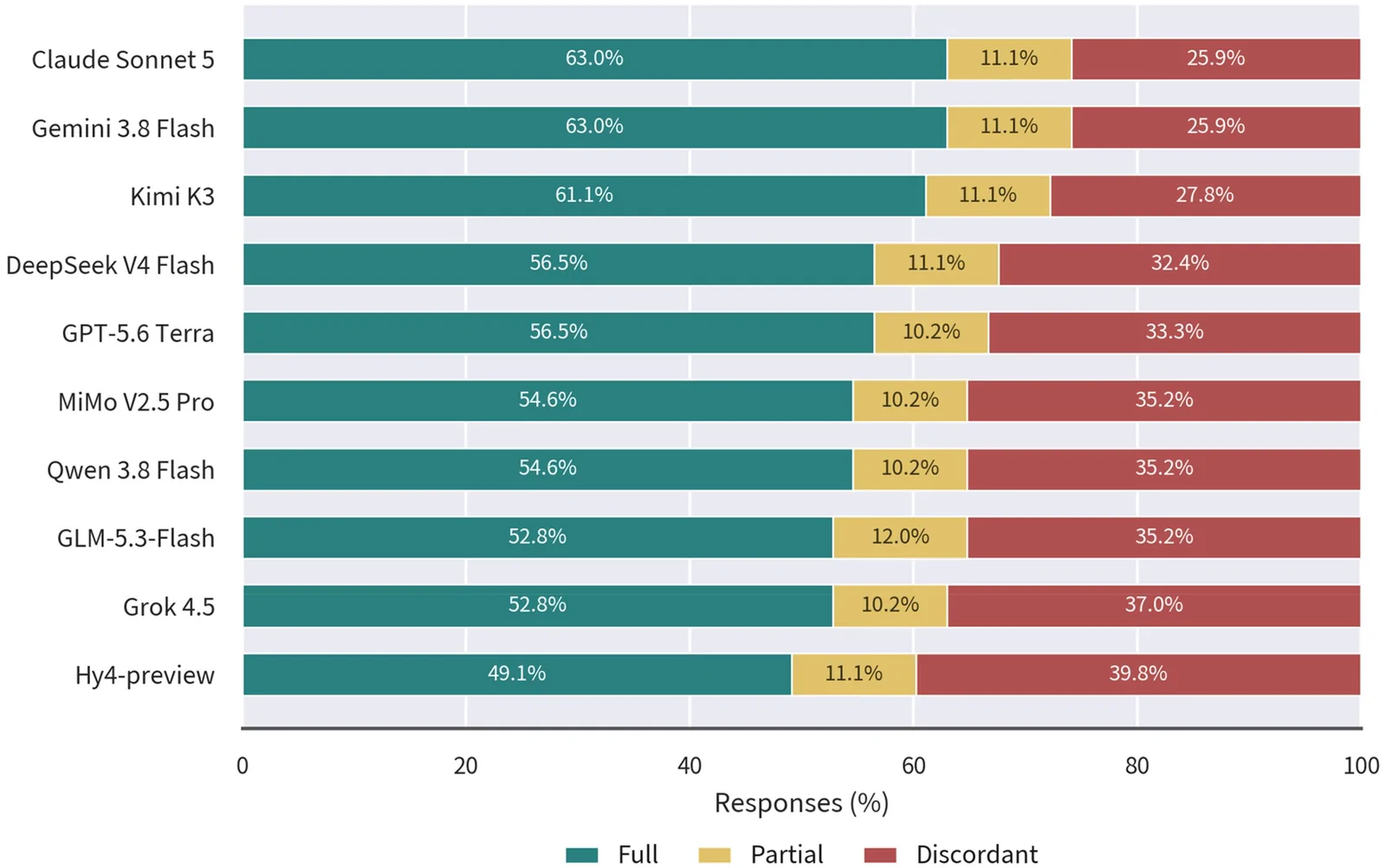
Model performance overview under structured-field scoring. Stacked bars show, for each model, the percentage of its 108 primary responses (36 primary scenarios   ×   3 runs) classified as Full, Partial, or Discordant under prompt-aligned structured-field scoring; models are ordered by Full concordance, and percentages are printed within each segment. Full concordance ranged from 49.1% to 63.0% across models, with wide and overlapping scenario-clustered confidence intervals (Table 1) and no significant overall model effect (Friedman *P* = .104; clustered GEE *P* = .186).

**Table 1:** Model-specific full concordance under the primary and semantic scoring frameworks.

Model	Structured Full, *n*/*N*; % (95% CI)	Contradiction-only Full, *n*/*N*; % (95% CI)	Full-semantic Full, *n*/*N*; % (95% CI)
DeepSeek V4 Flash	61/108; 56.5% (40.7–72.2)	60/108; 55.6% (39.8–70.4)	51/108; 47.2% (31.5–63.0)
GLM-5.3-Flash	57/108; 52.8% (38.0–67.6)	56/108; 51.9% (37.0–65.7)	42/108; 38.9% (24.1–53.7)
Claude Sonnet 5	68/108; 63.0% (47.2–77.8)	68/108; 63.0% (47.2–77.8)	56/108; 51.9% (36.1–68.5)
Gemini 3.8 Flash	68/108; 63.0% (47.2–77.8)	68/108; 63.0% (47.2–77.8)	56/108; 51.9% (36.1–67.6)
GPT-5.6 Terra	61/108; 56.5% (40.7–70.4)	61/108; 56.5% (40.7–70.4)	47/108; 43.5% (28.7–58.3)
Grok 4.5	57/108; 52.8% (37.0–68.5)	57/108; 52.8% (37.0–68.5)	45/108; 41.7% (25.9–57.4)
Qwen 3.8 Flash	59/108; 54.6% (38.0–70.4)	59/108; 54.6% (38.0–70.4)	44/108; 40.7% (25.0–57.4)
Hy4-preview	53/108; 49.1% (33.3–64.8)	53/108; 49.1% (33.3–64.8)	47/108; 43.5% (27.8–59.3)
Kimi K3	66/108; 61.1% (46.3–75.9)	66/108; 61.1% (46.3–75.9)	54/108; 50.0% (34.3–65.7)
MiMo V2.5 Pro	59/108; 54.6% (38.9–70.4)	59/108; 54.6% (38.9–70.4)	49/108; 45.4% (28.7–62.0)

The contradiction-only semantic analysis produced nearly identical absolute performance: 607 responses (56.2%) were Full, 112 (10.4%) Partial, and 361 (33.4%) Discordant. Only nine of the 125 downgrades identified under the full-semantic framework met the contradiction-only criterion: seven categorical overstatements of highly selected upfront CRS as standard care and two statements bundling IPCT with CRS as standard treatment. Overall between-model heterogeneity remained non-significant (Friedman chi-square = 15.50, df = 9, *P* = .078; clustered GEE Wald chi-square = 11.91, df = 9, *P* = .218; minimum Holm-adjusted pairwise *P* = .563).

Under the stricter full-semantic analysis, 491 responses (45.5%) were Full, 228 (21.1%) Partial, and 361 (33.4%) Discordant. The 125 semantic downgrades comprised 60 for incomplete standard surveillance, 23 for incomplete adjuvant duration or sequence, 14 for omission of a potential rectal-radiotherapy role, 10 for failure to state the approximately six-month systemic plan explicitly, nine for omission of reassessment after continued systemic therapy, seven categorical overstatements of upfront CRS, and two CRS-IPCT bundling statements. Full-semantic concordance ranged from 38.9% to 51.9%. The Friedman test reached nominal significance (chi-square = 18.51, df = 9, *P* = .030), but no pairwise comparison remained significant after Holm correction (minimum adjusted *P* = .381), and the clustered GEE model effect was not significant (Wald chi-square = 8.56, df = 9, *P* = .479). Thus, the scoring framework materially affected absolute concordance but did not establish a robust between-model hierarchy.

Confidence intervals are scenario-clustered bootstrap intervals based on 10,000 resamples. The structured analysis is the primary reporting endpoint; contradiction-only and full-semantic analyses are sensitivity analyses.

### 3.4 Primary-site and prompt-development sensitivity analyses

Excluding the single rectal-specific primary scenario (GRY-08) did not materially alter inference. Under structured scoring, the Friedman test was not significant (*P* = .078); under full-semantic scoring the Friedman result remained *P* = .030, with the clustered analysis continuing to provide no robust evidence of model separation. Because 14 structured-Full GRY-08 responses had been downgraded solely for omission of a potential rectal-radiotherapy role, this analysis also confirmed that the main semantic findings were not driven entirely by the rectal-specific item.

Excluding all 10 prompt-development scenarios reduced the analysis to 26 independent scenarios and 780 responses. Structured scoring then classified 393 responses (50.4%) as Full, 87 (11.2%) Partial, and 300 (38.5%) Discordant; between-model differences remained non-significant (Friedman chi-square = 12.30, *P* = .197; clustered GEE Wald chi-square = 13.25, *P* = .152). Contradiction-only Full concordance was also 50.4%. Full-semantic scoring classified 286 responses (36.7%) as Full, 187 (24.0%) Partial, and 307 (39.4%) Discordant; the corresponding Friedman *P* value was.053 and clustered GEE *P* = .091. Excluding development-overlapping scenarios therefore lowered absolute concordance estimates but did not change the inferential conclusion regarding the absence of a robust model hierarchy.

### 3.5 Repeatability, counterfactual robustness, and metachronous risk translation

Exact four-field self-consistency varied from 27.1% to 85.4% across models, with wide confidence intervals. High repeatability did not necessarily coincide with high consensus concordance. Run-level descriptive ADCR ranged from 0% to 50.0%. Because only six independent counterfactual pairs were available, pair-level majority success was used for precision estimates and ranged from 0 of 6 to 3 of 6 pairs; all Wilson confidence intervals were wide (Table 2).

**Table 2:** Repeatability, counterfactual robustness, metachronous risk translation, and certainty discrimination.

Model	Exact consistency, % (95% CI)	Pair-level ADCR, *n*/6; % (95% CI)	RRR, % (95% CI)	RMR, % (95% CI)	Certainty AUROC (95% CI)
DeepSeek V4 Flash	83.3 (70.4–91.3)	1/6; 16.7 (3.0–56.4)	81.5 (59.3–100.0)	37.5 (12.5–75.0)	0.618 (0.445–0.780)
GLM-5.3-Flash	50.0 (36.4–63.6)	0/6; 0.0 (0.0–39.0)	88.9 (77.8–100.0)	12.5 (0.0–29.2)	0.664 (0.483–0.828)
Claude Sonnet 5	77.1 (63.5–86.7)	2/6; 33.3 (9.7–70.0)	55.6 (22.2–88.9)	50.0 (12.5–87.5)	0.663 (0.488–0.827)
Gemini 3.8 Flash	66.7 (52.5–78.3)	3/6; 50.0 (18.8–81.2)	70.4 (44.4–92.6)	41.7 (12.5–75.0)	0.728 (0.575–0.867)
GPT-5.6 Terra	27.1 (16.6–41.0)	1/6; 16.7 (3.0–56.4)	66.7 (37.0–92.6)	29.2 (8.3–58.3)	0.764 (0.608–0.888)
Grok 4.5	50.0 (36.4–63.6)	0/6; 0.0 (0.0–39.0)	66.7 (40.7–88.9)	29.2 (4.2–62.5)	0.798 (0.671–0.901)
Qwen 3.8 Flash	68.8 (54.7–80.1)	2/6; 33.3 (9.7–70.0)	44.4 (11.1–77.8)	37.5 (12.5–75.0)	0.748 (0.618–0.868)
Hy4-preview	85.4 (72.8–92.8)	1/6; 16.7 (3.0–56.4)	77.8 (44.4–100.0)	16.7 (0.0–41.7)	0.712 (0.574–0.826)
Kimi K3	52.1 (38.3–65.5)	3/6; 50.0 (18.8–81.2)	92.6 (81.5–100.0)	33.3 (8.3–66.7)	0.703 (0.550–0.839)
MiMo V2.5 Pro	83.3 (70.4–91.3)	2/6; 33.3 (9.7–70.0)	88.9 (66.7–100.0)	41.7 (12.5–75.0)	0.602 (0.418–0.771)

Exact-consistency and pair-level ADCR intervals are Wilson intervals. RRR and RMR intervals are scenario-clustered bootstrap intervals. AUROC intervals are scenario-level bootstrap intervals. ADCR, actionable directional change rate; RRR, risk recognition rate; RMR, risk-management response rate.

Metachronous risk recognition also showed substantial imprecision. RRR ranged from 44.4% to 92.6% across nine independent risk-scored scenarios, whereas RMR ranged from 12.5% to 50.0% across eight high-risk scenarios. Correct classification therefore did not reliably translate into the required management direction; for example, GLM-5.3-Flash recognized metachronous risk in 88.9% of responses but achieved an RMR of 12.5%.

### 3.6 Self-reported certainty and expert-consensus alignment

Under full-semantic scoring, mean certainty was 87.9 for Full responses, 86.7 for Partial responses, and 82.8 for Discordant responses; corresponding medians were 90, 89, and 82. After adjustment for model and run with scenario-clustered standard errors, certainty was 5.47 points higher for Full than Discordant responses (*P* < .001) and 4.31 points higher for Partial than Discordant responses (*P* = .002). Nevertheless, 101 of 361 Discordant responses (28.0%) had certainty of at least 90. Model-specific AUROCs for distinguishing Full from non-Full responses ranged from 0.602 to 0.798, with broad scenario-bootstrap confidence intervals (Table 2).

Among 17 independently voted consensus blocks represented in the primary benchmark, Delphi round-2 agreement showed a moderate but non-significant association with mean model certainty (Spearman rho=0.441, *P* = .076) and no association with full-semantic Full-concordance rate (rho=-0.033, *P* = .901).

### 3.7 Clinically consequential discordance flags

Twenty-one of 1,080 primary responses (1.9%) met a prespecified clinically consequential discordance flag, but these 21 responses arose from only five of the 36 independent primary scenarios (13.9%; Wilson 95% CI 6.1%–28.7%). Incorrect treatment sequence accounted for nine flagged responses, categorical overstatement of a conditional recommendation for seven, and undertreatment for five (Figure 3). No response met the prespecified definitions for overtreatment, failure to recognize unresectability, or inappropriate IPCT. Nine of the 21 flagged responses had self-reported certainty of at least 90. These events were treated as clustered benchmark observations rather than independent clinical safety events (Table 3).

**Figure 3:**
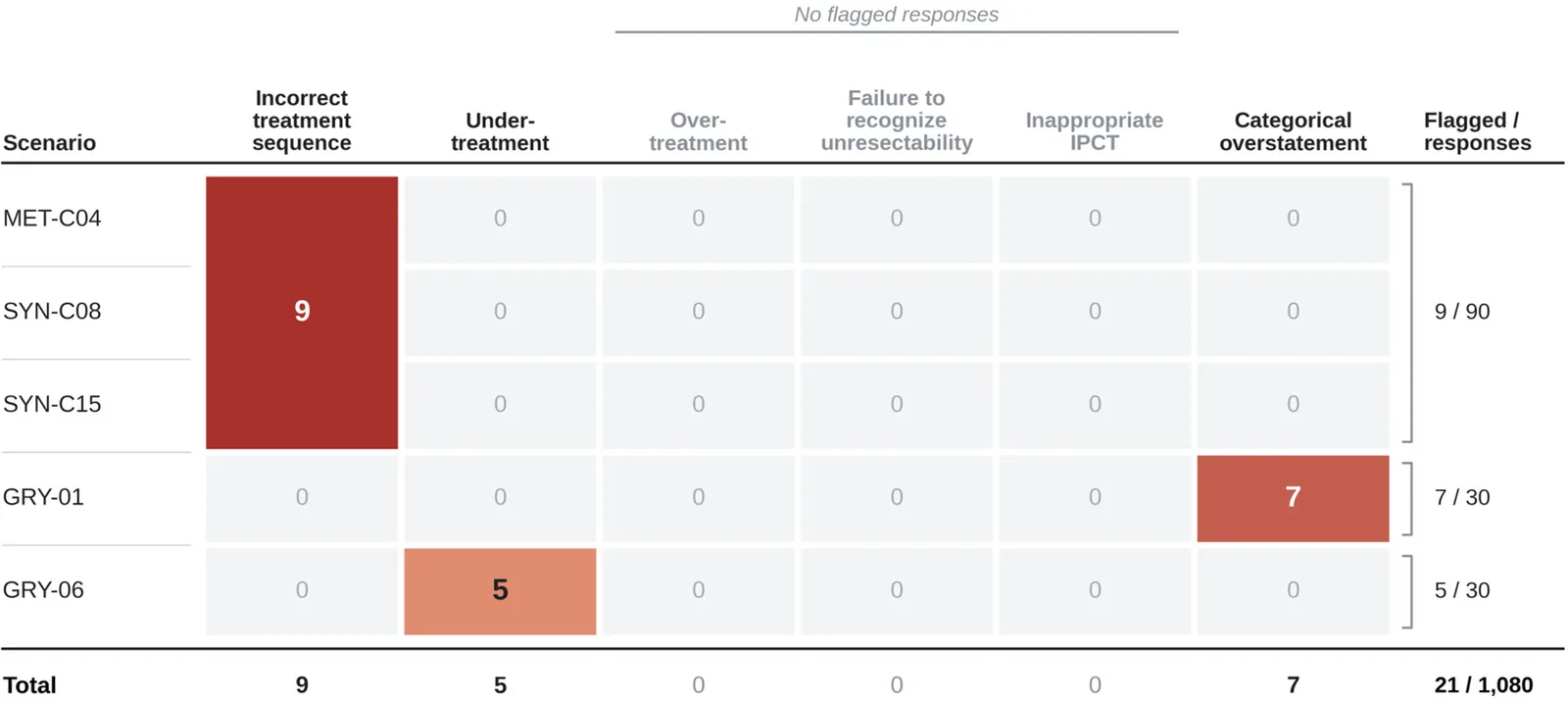
Heatmap of clinically consequential discordance flags among primary responses. Cells show the number of flagged responses for each scenario and prespecified flag category; the right-hand column gives flagged responses over total responses per scenario (10 models   ×   3 runs = 30 responses per scenario). Twenty-one of 1,080 primary responses (1.9%) arose from five of 36 primary scenarios. The nine responses flagged for incorrect treatment sequence were distributed across MET-C04, SYN-C08, and SYN-C15 and are shown pooled (9/90). Flagged responses in GRY-01 were categorical overstatements of highly selected upfront CRS as standard, routine, or mandatory care (7/30), and those in GRY-06 were undertreatment, with postoperative systemic therapy omitted after complete upfront CRS and surveillance selected instead (5/30). No response met the prespecified definitions for overtreatment, failure to recognize unresectability, or inappropriate IPCT. Flags are descriptive benchmark events, not patient-level safety outcomes. CRS, cytoreductive surgery; IPCT, intraperitoneal chemotherapy.

**Table 3:** Prespecified clinically consequential discordance patterns.

Category	Flagged responses, *n*	Independent scenarios	Observed pattern
Incorrect treatment sequence	9	3 (MET-C04, SYN-C08, SYN-C15)	Treatment selected at the wrong stage, including CRS despite high-risk timing, first-line progression, or signet-ring sequencing.
Categorical overstatement	7	1 (GRY-01)	Highly selected upfront CRS described as standard, routine, or mandatory care.
Undertreatment	5	1 (GRY-06)	Postoperative systemic therapy omitted after complete upfront CRS, with surveillance selected instead.

## 4 Discussion

In the primary analysis, prompt-aligned structured Full concordance across 10 contemporary LLMs ranged from 49.1% to 63.0%, with 56.4% of all primary responses classified as Full. Neither the scenario-level Friedman test nor the clustered GEE analysis supported a significant overall model effect, and no pairwise comparison remained significant after multiplicity adjustment. The contradiction-only semantic analysis produced almost identical absolute performance, whereas the omission-sensitive full-semantic framework reduced overall Full concordance to 45.5% and yielded a nominally significant Friedman test without corroboration from pairwise or clustered analyses. The central inference is therefore not that one model consistently outperformed the others, but that absolute apparent performance depended materially on how semantic completeness was defined while the evidence for a stable between-model hierarchy remained weak.

This distinction addresses an important benchmarking problem. The frozen prompt asked for a single next action, structured CRS/IPCT/risk fields, and a rationale of at most 50 words. Most full-semantic downgrades reflected omissions of additional surveillance, treatment-duration, reassessment, or rectal-radiotherapy detail that the prompt did not explicitly require. When semantic downgrading was restricted to explicit contradictions, Full concordance returned to 56.2%, nearly identical to structured scoring. Structured-only benchmarking can therefore miss clinically meaningful contradictory language, but an omission-sensitive rubric can also penalize models for information outside the explicitly requested response format. Reporting both prompt-aligned and stricter semantic results makes that dependence visible rather than embedding it in a single performance number.

The observed concordance remains broadly consistent with a growing literature showing substantial task dependence in oncologic LLM evaluation. Comparisons with multidisciplinary tumor boards have reported higher agreement in some breast-cancer and guideline-based rectal-cancer settings, whereas thoracic oncology studies have shown more modest single-session agreement (13, 14, 16, 17, 19). Prospective head and neck work has examined locally deployed LLMs as supplementary inputs to real tumor-board workflows (20). Sarcoma provides an especially relevant counterpoint: ChatGPT-4o showed heterogeneous performance across specialties in a multidisciplinary sarcoma-board simulation and a ring-trial comparison across 21 sarcoma centers found only partial alignment between four LLMs and human tumor-board recommendations (25, 26). These differences reinforce that model performance cannot be generalized across tumor types, decision granularity, or benchmark construction.

Repeatability and correctness were also distinct. Exact four-field consistency ranged from 27.1% to 85.4%, and the most internally stable models were not necessarily those with the highest consensus concordance. This pattern is consistent with work showing that repeatability and diagnostic or decision accuracy can be only loosely related properties of LLM behavior (27). Stable repetition of a wrong or incompletely contextualized decision is not evidence of clinical reliability.

Counterfactual testing exposed a second limitation. Run-level ADCR never exceeded 50%, and when the six counterfactual pairs were treated as the independent units, no model was directionally successful in more than three of six pairs with a majority of runs. The resulting confidence intervals were necessarily very wide. Each pair changed a single decisive feature, such as performance status, cytoreduction feasibility, progression after first-line therapy, liver-metastasis burden, signet-ring histology, or sidedness. These results suggest that ordinary concordance on static vignettes may coexist with incomplete sensitivity to the specific variable that should change management.

A related dissociation emerged in metachronous disease. RRR reached 92.6% for one model, yet RMR never exceeded 50.0%, and GLM-5.3-Flash recognized risk in 88.9% of responses while translating that recognition into an acceptable systemic/next-line management response in only 12.5%. Correct classification of a risk feature therefore did not guarantee an appropriate downstream action. This distinction is particularly relevant in CRC-PM, where the consensus pathway links disease-free interval, nodal status, grade, histology, PCI, and sidedness to treatment sequencing rather than treating risk classification as an isolated endpoint.

Self-reported certainty offered limited protection against these failures. Although certainty was statistically higher for Full than Discordant responses, 28.0% of full-semantic Discordant responses carried certainty of at least 90, and model-specific certainty AUROCs had broad confidence intervals (22–24). High-certainty discordance creates a plausible risk of automation bias if fluent recommendations are accepted without scrutiny. The present study did not measure automation bias directly, but the combination of confident discordance and incomplete counterfactual responsiveness provides a rationale for testing human factors explicitly in future work.

The 21 prespecified clinically consequential discordance flags should also be interpreted conservatively. They arose from only five independent scenarios, so counting each response as a separate clinical event would overstate the evidence. The recurrent patterns nevertheless identify where benchmark failures concentrated: treatment sequencing after progression or adverse histology, postoperative therapy after upfront CRS, and categorical overstatement of a highly selected surgical strategy. These are clinically important decision nodes in the updated consensus framework, but disagreement with one consensus-derived reference is not equivalent to observed patient harm.

The study evaluated LLMs in isolation and therefore cannot determine whether incorporating model output into clinician or multidisciplinary-team workflows improves decision quality. The data do not establish benefit from supervised adjunctive use, nor do they support autonomous use for CRS/IPCT candidacy or treatment sequencing. Prospective clinician-plus-LLM studies with patient-relevant endpoints are required to test whether model assistance improves, worsens, or leaves unchanged real-world decisions.

Several additional risks should be considered before extrapolating benchmark performance to clinical use. General-purpose LLMs may generate factually incorrect or fabricated information, cite nonexistent or inappropriate sources, rely on outdated knowledge, or provide plausible recommendations without transparent provenance. Outputs may vary across repeated queries, model updates, or interface configurations, complicating reproducibility and accountability. In patient-facing settings, confident but incorrect recommendations could encourage inappropriate self-management or delay consultation with the treating oncology team. Externally hosted systems also raise confidentiality and data-protection concerns when identifiable clinical information is entered into third-party platforms, while legal and professional responsibility for decisions influenced by probabilistic model output remains incompletely defined (28). These risks are particularly relevant in oncology, where treatment sequence and resectability decisions may be difficult to reverse.

Whether guideline-grounded technical architectures can improve performance remains an open question. Retrieval-augmented or rule-constrained systems have improved guideline adherence in other high-stakes decision-support tasks, and analogous CRC-PM systems may outperform the single frozen non-retrieval prompt evaluated here (29, 30). Such systems should still be assessed for version stability, source provenance, counterfactual behavior, clinician interaction, and patient outcomes rather than assumed to be safer because retrieval is present.

Several limitations should be considered. First, the benchmark used synthetic decision-isolation vignettes rather than complete patient records. Many demographic, staging, molecular, imaging, and regimen-level variables were intentionally unspecified unless directly relevant to the tested node, and the method by which peritoneal metastases were established was generally not specified. Second, although the final reference classifications were independently validated by two blinded colorectal surgeons with high inter-rater agreement, residual subjectivity remains inherent in translating pathway-level consensus into atomic decisions. Third, 10 scenarios were used during prompt development and retained in the formal benchmark. Excluding all overlapping cases reduced absolute concordance but did not materially change the inferential conclusion. Fourth, the scoring framework itself influenced absolute performance: structured and contradiction-only results were substantially higher than omission-sensitive full-semantic results. Fifth, the effective sample comprised only 36 independent primary scenarios, with 26 remaining after development-case exclusion; counterfactual and metachronous secondary analyses relied on only six, nine, or eight independent units and therefore had wide confidence intervals. Sixth, testing occurred at a single time point in September 2026; model behavior may change with version updates. Seventh, the study used a single zero-shot prompt without retrieval, multi-turn clarification, or multimodal input. Finally, no clinician-plus-LLM arm or patient outcome was studied, so neither clinical safety nor benefit from supervised use can be inferred.

## 5 Conclusion

In this exploratory benchmark, 10 contemporary LLMs showed moderate prompt-aligned concordance with an expert-consensus CRC-PM reference framework, and apparent performance changed substantially when stricter semantic completeness rules were applied. Independent expert validation supported reproducibility of the reference classifications, but wide scenario-level confidence intervals, weak counterfactual responsiveness, incomplete translation of risk recognition into management, and high-certainty discordance limited the strength of model comparisons. The data did not establish a stable between-model hierarchy and cannot determine whether clinician-supervised LLM use improves care. Future evaluation should prioritize prospective human-AI collaboration, guideline-grounded systems, transparent model provenance, and patient-relevant outcomes.

## Data Availability

The original contributions presented in the study are included in the article/Supplementary Material, further inquiries can be directed to the corresponding author/s.
