# Supplementary material for: Large language models for complex decision-making in colorectal cancer with peritoneal metastases
Source: Front Med (Lausanne). 2026 Oct 28;13:1997728. doi: 10.3389/fmed.2026.1997728 (PMC13751452; doi:10.3389/fmed.2026.1997728)
Supplement: Supplementary file 1 [file Table1.docx]

**Supplementary Materials**

**Large Language Models for Complex Decision-Making in Colorectal Cancer With Peritoneal Metastases**

Supplementary Methods, Tables, Benchmark Vignettes, Scoring Rules, and Validation Analyses

# Contents

- Supplementary Methods
- Supplementary Table S1. Case-level vignette characteristics and availability of requested clinical variables
- Supplementary Table S2. Model aliases, access routes, API-returned identifiers, and inference settings
- Supplementary Table S3. Prompt-development history and frozen V3 prompt
- Supplementary Table S4. Precision analyses for secondary outcomes and high-certainty discordance
- Supplementary Table S5. Sensitivity analyses excluding the rectal-specific and prompt-development scenarios
- Supplementary Table S6. Independent expert validation of the benchmark reference standard
- Supplementary Table S7. Full benchmark vignette set and reference classifications
- Supplementary Table S8. Case-specific scoring rules and prespecified clinically consequential discordance triggers
- Supplementary Table S9. Semantic adjudication downgrades and clinically consequential discordance taxonomy

# Supplementary Methods

## Benchmark construction and clinical definitions

The benchmark was derived from the pathway-based expert consensus framework for colorectal cancer with peritoneal metastases (CRC-PM) reported by Schultz et al. The final benchmark comprised 48 synthetic decision-isolation vignettes: 28 core cases, 8 gray-zone cases, and 12 counterfactual cases forming 6 paired perturbations. Thirty-six non-counterfactual scenarios constituted the primary concordance set; the 12 counterfactual scenarios were analyzed separately for directional robustness.

Synchronous peritoneal metastases were operationally defined as peritoneal disease present at the initial colorectal cancer presentation. Metachronous peritoneal metastases referred to peritoneal disease detected after previous resection of the primary colorectal tumor. Within the metachronous pathway, a disease-free interval of <12 months was treated as a high-risk feature. Where systemic-therapy duration was relevant to the tested decision node, the reference framework used either 3-6 months of systemic therapy before reassessment or completion of approximately six months of total systemic therapy when preoperative treatment had already been given.

The vignettes were designed to isolate individual decision nodes rather than to reproduce complete patient charts. Clinical characteristics that were not explicitly stated in a vignette were not inferred, imputed, or supplied to the models. Supplementary Table S1 therefore records unstated variables as 'Not specified'.

## Prompt development and formal model testing

Prompt development proceeded in four stages before the formal multi-model benchmark was frozen. The source consensus pathways were first decomposed into actionable management nodes. A structured zero-shot prompt was then developed to request a single next management action together with CRS, IPCT, metachronous-risk, certainty, and brief-rationale fields. The benchmark was subsequently expanded to include core, gray-zone, and counterfactual scenarios, and a 10-case DeepSeek pilot using SYN-C01 through SYN-C10 was used to identify formatting and scenario-specific interpretive ambiguity. Pilot-informed revisions were incorporated into Benchmark V2 and the final V3 prompt before formal multi-model testing. Development/test overlap was addressed by a sensitivity analysis excluding SYN-C01-SYN-C10.

Formal testing was conducted on September 8-9, 2026. Ten model aliases were evaluated three times per vignette, producing 1,440 schema-valid responses after replacement of 14 initial schema- or parsing-invalid responses under identical settings. The frozen prompt and benchmark hashes recorded in the formal logs were invariant across model runs (system prompt SHA-256: 3425a03d5cdda68d40e3c66a7c343939c380f56352a2b078c93e2e5b33dd2142; benchmark SHA-256: 395f0d320ac604b0bcad2d51983255e0b9ddfa00a2354ff7a657bbff43210d67).

## Scoring framework

The revised primary reporting endpoint was prompt-aligned structured-field concordance. Only fields relevant to the decision node were scored. A response was classified as Full when all scored structured fields matched their prespecified acceptable values; Partial when at least one scored field was conditionally acceptable without a directly discordant field; and Discordant when at least one scored field was incompatible with the reference decision.

Two semantic sensitivity analyses were performed. Under the contradiction-only rule, a structured grade could be downgraded only for an explicit clinically meaningful contradiction, an incorrect assertion of standard care, or a categorical overstatement of a conditional recommendation. The stricter full-semantic analysis retained the original case-specific omission-sensitive semantic rules. Semantic adjudication could downgrade but not upgrade a structured grade.

Secondary outcomes were exact four-field self-consistency across three repeated runs, actionable directional change rate (ADCR) in six counterfactual pairs, metachronous risk recognition rate (RRR), risk-management response rate (RMR), self-reported certainty discrimination, high-certainty discordance, and prespecified clinically consequential discordance flags. The flags were descriptive benchmark events rather than patient-level safety outcomes.

## Independent expert validation

Two attending colorectal surgeons, each with 5 years of clinical experience, independently reviewed all 48 vignettes and the corresponding reference classifications while blinded to model identity and model-generated responses. Agreement was evaluated separately for primary management action, CRS position, IPCT position, and metachronous risk classification using raw agreement and Cohen's kappa. Exact four-domain agreement was also calculated. Disagreements were adjudicated after independent review.

## Statistical precision

The 36 primary scenarios, rather than the 108 repeated responses per model, were treated as the independent clinical units. The scenario count was determined by coverage of clinically distinct consensus decision nodes rather than by a formal power calculation. With 36 independent scenarios, a proportion near 0.50 has a nominal 95% Wilson interval half-width of approximately 15.5 percentage points. Scenario-clustered bootstrap intervals preserved all three runs within each resampled scenario. Wilson intervals were used for exact self-consistency and pair-level majority ADCR. RRR, RMR, and AUROC precision estimates used scenario-level resampling. The study was interpreted as exploratory.

**Supplementary Table S1. Case-level vignette characteristics and availability of requested clinical variables**

Variables not explicitly stated in the frozen vignette are reported as 'Not specified'. No values were retrospectively imputed.

**Panel A.** Demographic, performance, staging, and disease-burden characteristics

| **Case ID** | **Primary-site specificity** | **Age** | **Sex** | **Performance status / ECOG** | **T category** | **N category** | **PCI** |
| --- | --- | --- | --- | --- | --- | --- | --- |
| SYN-C01 | Generic CRC / primary site not specified | Not specified | Not specified | Not specified | Not specified | Not specified | Not specified |
| SYN-C02 | Generic CRC / primary site not specified | Not specified | Not specified | Poor performance status; high-risk surgical candidate | Not specified | Not specified | Not specified |
| SYN-C03 | Generic CRC / primary site not specified | Not specified | Not specified | Medically fit | Not specified | Not specified | Not specified |
| SYN-C04 | Generic CRC / primary site not specified | Not specified | Not specified | Medically fit | Not specified | Not specified | Not specified |
| SYN-C05 | Generic CRC / primary site not specified | Not specified | Not specified | Not specified | Not specified | Not specified | Not specified |
| SYN-C06 | Generic CRC / primary site not specified | Not specified | Not specified | Not specified | Not specified | Not specified | Not specified |
| SYN-C07 | Generic CRC / primary site not specified | Not specified | Not specified | Not specified | Not specified | Not specified | Not specified |
| SYN-C08 | Generic CRC / primary site not specified | Not specified | Not specified | Not specified | Not specified | Not specified | Not specified |
| SYN-C09 | Generic CRC / primary site not specified | Not specified | Not specified | Not specified | Not specified | Not specified | Not specified |
| SYN-C10 | Generic CRC / primary site not specified | Not specified | Not specified | Remains fit for surgery | Not specified | Not specified | Low (qualitative) |
| SYN-C11 | Generic CRC / primary site not specified | Not specified | Not specified | Not specified | Not specified | Not specified | Not specified |
| SYN-C12 | Generic CRC / primary site not specified | Not specified | Not specified | Fit | Not specified | Not specified | 12 |
| SYN-C13 | Generic CRC / primary site not specified | Not specified | Not specified | Fit | Not specified | Not specified | Not specified |
| SYN-C14 | Generic CRC / primary site not specified | Not specified | Not specified | Fit | Not specified | Not specified | Not specified |
| SYN-C15 | Generic CRC / primary site not specified | Not specified | Not specified | Fit | Not specified | Not specified | Low (qualitative) |
| MET-C01 | Generic CRC / primary site not specified | Not specified | Not specified | Not specified | Not specified | Not specified | Not specified |
| MET-C02 | Generic CRC / primary site not specified | Not specified | Not specified | Poor functional status | Not specified | Not specified | Not specified |
| MET-C03 | Colon sidedness specified | Not specified | Not specified | Fit | Not specified | N0 / node-negative stated | Low (qualitative) |
| MET-C04 | Generic CRC / primary site not specified | Not specified | Not specified | Fit | Not specified | Not specified | Not specified |
| MET-C05 | Generic CRC / primary site not specified | Not specified | Not specified | Fit | Not specified | Node-positive; exact N category not specified | Not specified |
| MET-C06 | Generic CRC / primary site not specified | Not specified | Not specified | Fit | Not specified | Not specified | Not specified |
| MET-C07 | Generic CRC / primary site not specified | Not specified | Not specified | Fit | Not specified | Not specified | Not specified |
| MET-C08 | Generic CRC / primary site not specified | Not specified | Not specified | Fit | Not specified | Not specified | High (qualitative) |
| MET-C09 | Colon sidedness specified | Not specified | Not specified | Fit | Not specified | Not specified | Not specified |
| MET-C10 | Generic CRC / primary site not specified | Not specified | Not specified | Not specified | Not specified | Not specified | Not specified |
| MET-C11 | Generic CRC / primary site not specified | Not specified | Not specified | Not specified | Not specified | Not specified | Not specified |
| MET-C12 | Generic CRC / primary site not specified | Not specified | Not specified | Not specified | Not specified | Not specified | Low (qualitative) |
| MET-C13 | Generic CRC / primary site not specified | Not specified | Not specified | Not specified | Not specified | Not specified | Not specified |
| GRY-01 | Generic CRC / primary site not specified | Not specified | Not specified | Fit; ECOG 0 | Not specified | Not specified | Low (qualitative) |
| GRY-02 | Generic CRC / primary site not specified | Not specified | Not specified | Fit | Not specified | Not specified | Not specified |
| GRY-03 | Generic CRC / primary site not specified | Not specified | Not specified | Not specified | Not specified | Not specified | Not specified |
| GRY-04 | Generic CRC / primary site not specified | Not specified | Not specified | Not specified | Not specified | Not specified | Not specified |
| GRY-05 | Generic CRC / primary site not specified | Not specified | Not specified | Not specified | Not specified | Not specified | Not specified |
| GRY-06 | Generic CRC / primary site not specified | Not specified | Not specified | Not specified | Not specified | Not specified | Not specified |
| GRY-07 | Generic CRC / primary site not specified | Not specified | Not specified | Not specified | Not specified | Not specified | Not specified |
| GRY-08 | Rectal cancer specified | Not specified | Not specified | Medically fit | Not specified | Not specified | Not specified |
| CF01-A | Generic CRC / primary site not specified | 58 | Not specified | ECOG 0 | Not specified | Not specified | Low (qualitative) |
| CF01-B | Generic CRC / primary site not specified | 58 | Not specified | ECOG 3 | Not specified | Not specified | Low (qualitative) |
| CF02-A | Generic CRC / primary site not specified | Not specified | Not specified | Not specified | Not specified | Not specified | Not specified |
| CF02-B | Generic CRC / primary site not specified | Not specified | Not specified | Not specified | Not specified | Not specified | Not specified |
| CF03-A | Generic CRC / primary site not specified | Not specified | Not specified | Not specified | Not specified | Not specified | Not specified |
| CF03-B | Generic CRC / primary site not specified | Not specified | Not specified | Not specified | Not specified | Not specified | Not specified |
| CF04-A | Generic CRC / primary site not specified | Not specified | Not specified | Fit | Not specified | Not specified | 12 |
| CF04-B | Generic CRC / primary site not specified | Not specified | Not specified | Fit | Not specified | Not specified | 12 |
| CF05-A | Generic CRC / primary site not specified | Not specified | Not specified | Fit; ECOG 0 | Not specified | Not specified | Low (qualitative) |
| CF05-B | Generic CRC / primary site not specified | Not specified | Not specified | Fit; ECOG 0 | Not specified | Not specified | Low (qualitative) |
| CF06-A | Colon sidedness specified | Not specified | Not specified | Fit | Not specified | N0 / node-negative stated | Low (qualitative) |
| CF06-B | Colon sidedness specified | Not specified | Not specified | Fit | Not specified | N0 / node-negative stated | Low (qualitative) |

**Panel B.** Extraperitoneal disease, histology, molecular status, and sidedness

| **Case ID** | **Extraperitoneal disease** | **Histology / grade** | **Molecular status** | **Sidedness** |
| --- | --- | --- | --- | --- |
| SYN-C01 | Not specified | Colorectal adenocarcinoma | Not specified | Not specified |
| SYN-C02 | Not specified | Not specified | Not specified | Not specified |
| SYN-C03 | Present: extensive unresectable solid-organ/extraperitoneal metastases | Not specified | Not specified | Not specified |
| SYN-C04 | No unresectable extraperitoneal disease stated | Not specified | Not specified | Not specified |
| SYN-C05 | Not specified | Not specified | Not specified | Not specified |
| SYN-C06 | Not specified | Not specified | Not specified | Not specified |
| SYN-C07 | Not specified | Not specified | Not specified | Not specified |
| SYN-C08 | Not specified | Not specified | Not specified | Not specified |
| SYN-C09 | Not specified | Not specified | Not specified | Not specified |
| SYN-C10 | Absent stated | Not specified | Not specified | Not specified |
| SYN-C11 | Not specified | Not specified | Not specified | Not specified |
| SYN-C12 | Present: 2 technically resectable liver metastases | Not specified | Not specified | Not specified |
| SYN-C13 | Present: 5 liver metastases | Not specified | Not specified | Not specified |
| SYN-C14 | Ovarian Krukenberg metastasis; no other prohibitive factor stated | Not specified | Not specified | Not specified |
| SYN-C15 | Isolated PM stated | Poorly differentiated signet-ring cell carcinoma | Not specified | Not specified |
| MET-C01 | Not specified | Not specified | Not specified | Not specified |
| MET-C02 | Present: unresectable extraperitoneal metastatic disease | Not specified | Not specified | Not specified |
| MET-C03 | Isolated PM stated | Low grade; no signet-ring histology | Not specified | Left-sided |
| MET-C04 | Isolated PM stated | Not specified | Not specified | Not specified |
| MET-C05 | Isolated PM stated | Not specified | Not specified | Not specified |
| MET-C06 | Isolated PM stated | High grade | Not specified | Not specified |
| MET-C07 | Isolated PM stated | Signet-ring cell histology | Not specified | Not specified |
| MET-C08 | Isolated PM stated | Not specified | Not specified | Not specified |
| MET-C09 | Isolated PM stated | Not specified | Not specified | Right-sided |
| MET-C10 | Not specified | Not specified | Not specified | Not specified |
| MET-C11 | Not specified | Not specified | Not specified | Not specified |
| MET-C12 | Absent stated | Not specified | Not specified | Not specified |
| MET-C13 | Not specified | Not specified | Not specified | Not specified |
| GRY-01 | Isolated PM stated | No signet-ring histology | Not specified | Not specified |
| GRY-02 | Not specified | Not specified | Not specified | Not specified |
| GRY-03 | Not specified | Not specified | Not specified | Not specified |
| GRY-04 | Not specified | Not specified | Not specified | Not specified |
| GRY-05 | Not specified | Not specified | Not specified | Not specified |
| GRY-06 | Not specified | Not specified | Not specified | Not specified |
| GRY-07 | Not specified | Not specified | Not specified | Not specified |
| GRY-08 | No unresectable extraperitoneal disease stated | Rectal adenocarcinoma | Not specified | Rectal |
| CF01-A | Isolated PM stated | No signet-ring histology | Not specified | Not specified |
| CF01-B | Isolated PM stated | No signet-ring histology | Not specified | Not specified |
| CF02-A | Not specified | Not specified | Not specified | Not specified |
| CF02-B | Not specified | Not specified | Not specified | Not specified |
| CF03-A | Not specified | Not specified | Not specified | Not specified |
| CF03-B | Not specified | Not specified | Not specified | Not specified |
| CF04-A | Present: 2 technically resectable liver metastases | Not specified | Not specified | Not specified |
| CF04-B | Present: 5 liver metastases | Not specified | Not specified | Not specified |
| CF05-A | Isolated PM stated | Conventional moderately differentiated adenocarcinoma | Not specified | Not specified |
| CF05-B | Isolated PM stated | Signet-ring cell carcinoma | Not specified | Not specified |
| CF06-A | Isolated PM stated | Low grade; no signet-ring histology | Not specified | Left-sided |
| CF06-B | Isolated PM stated | Low grade; no signet-ring histology | Not specified | Right-sided |

**Panel C.** Peritoneal-metastasis ascertainment and systemic-therapy context

| **Case ID** | **PM establishment method** | **Systemic-therapy regimen** | **Line / stage** | **Duration** | **Response** |
| --- | --- | --- | --- | --- | --- |
| SYN-C01 | Staging confirmed PM; modality not specified | Not specified | Not specified | Not specified | Not specified |
| SYN-C02 | Not specified | Not specified | Not specified | Not specified | Not specified |
| SYN-C03 | Not specified | Not specified | Not specified | Not specified | Not specified |
| SYN-C04 | Not specified | Not specified | None yet | Not specified | Not specified |
| SYN-C05 | Not specified | Not specified | Initial systemic therapy completed; exact line not specified | Not specified | Not specified |
| SYN-C06 | Not specified | Not specified | First-line completed | Not specified | No disease progression |
| SYN-C07 | Not specified | Not specified | First-line completed | Not specified | No disease progression |
| SYN-C08 | Not specified | Not specified | First-line | Not specified | Progression |
| SYN-C09 | Not specified | Not specified | First-line | Not specified | Progression |
| SYN-C10 | Not specified | Not specified | Systemic therapy given; line not specified | Not specified | No progression |
| SYN-C11 | Not specified | Not specified | Not specified | Not specified | Not specified |
| SYN-C12 | Not specified | Not specified | Systemic therapy given; line not specified | Not specified | Not specified |
| SYN-C13 | Not specified | Not specified | Not specified | Not specified | Not specified |
| SYN-C14 | Not specified | Not specified | Not specified | Not specified | Not specified |
| SYN-C15 | Not specified | Not specified | Not specified | Not specified | Not specified |
| MET-C01 | Not specified | Not specified | Not specified | Not specified | Not specified |
| MET-C02 | Not specified | Not specified | Not specified | Not specified | Not specified |
| MET-C03 | Not specified | Not specified | Not specified | Not specified | Not specified |
| MET-C04 | Not specified | Not specified | Not specified | Not specified | Not specified |
| MET-C05 | Not specified | Not specified | Not specified | Not specified | Not specified |
| MET-C06 | Not specified | Not specified | Not specified | Not specified | Not specified |
| MET-C07 | Not specified | Not specified | Not specified | Not specified | Not specified |
| MET-C08 | Not specified | Not specified | Not specified | Not specified | Not specified |
| MET-C09 | Not specified | Not specified | Not specified | Not specified | Not specified |
| MET-C10 | Not specified | Not specified | None yet for metastatic recurrence | Not specified | Not specified |
| MET-C11 | Not specified | Not specified | Systemic therapy completed; line not specified | 3-6 months | Progression (radiographic and biomarker) |
| MET-C12 | Not specified | Not specified | Not specified | Not specified | Not specified |
| MET-C13 | Not specified | Not specified | Not specified | Not specified | Not specified |
| GRY-01 | Not specified | Not specified | Not specified | Not specified | Not specified |
| GRY-02 | Not specified | Not specified | Not specified | Not specified | Not specified |
| GRY-03 | Not specified | Not specified | Not specified | Not specified | Not specified |
| GRY-04 | Not specified | Not specified | Not specified | Not specified | Not specified |
| GRY-05 | Not specified | Not specified | Not specified | Not specified | Not specified |
| GRY-06 | Not specified | Not specified | No preoperative systemic therapy | Not specified | Not specified |
| GRY-07 | Not specified | Not specified | Preoperative systemic therapy; line not specified | 4 months total so far | Not specified |
| GRY-08 | Not specified | Not specified | None yet | Not specified | Not specified |
| CF01-A | Not specified | Not specified | Not specified | Not specified | Not specified |
| CF01-B | Not specified | Not specified | Not specified | Not specified | Not specified |
| CF02-A | Not specified | Not specified | First-line completed | Not specified | No progression |
| CF02-B | Not specified | Not specified | First-line completed | Not specified | No progression |
| CF03-A | Not specified | Not specified | First-line completed | Not specified | No progression |
| CF03-B | Not specified | Not specified | First-line completed | Not specified | Progression |
| CF04-A | Not specified | Not specified | Systemic therapy given; line not specified | Not specified | Not specified |
| CF04-B | Not specified | Not specified | Systemic therapy given; line not specified | Not specified | Not specified |
| CF05-A | Not specified | Not specified | Not specified | Not specified | Not specified |
| CF05-B | Not specified | Not specified | Not specified | Not specified | Not specified |
| CF06-A | Not specified | Not specified | Not specified | Not specified | Not specified |
| CF06-B | Not specified | Not specified | Not specified | Not specified | Not specified |

Abbreviations: ECOG, Eastern Cooperative Oncology Group; PCI, peritoneal cancer index; PM, peritoneal metastases.

**Supplementary Table S2. Model aliases, access routes, API-returned identifiers, and inference settings**

**Panel A.** Model identity and access route

| Model | Requested alias | API-returned identifier | Provider / interface | Testing date (UTC) |
| --- | --- | --- | --- | --- |
| DeepSeek V4 Flash | deepseek-v4-flash | deepseek-v4-flash | DeepSeek official API | 2026-09-08 |
| GLM-5.3-Flash | glm-5.3-flash | glm-5.3-flash | Zhipu GLM API | 2026-09-09 |
| Claude Sonnet 5 | claude-sonnet-5 | claude-sonnet-5 | Anthropic Claude API | 2026-09-09 |
| Gemini 3.8 Flash | gemini-3.8-flash | gemini-3.8-flash | Google Gemini API | 2026-09-09 |
| GPT-5.6 Terra | gpt-5.6-terra | gpt-5.6-terra | OpenAI API | 2026-09-09 |
| Grok 4.5 | grok-4.5 | grok-4.5 | xAI API | 2026-09-09 |
| Qwen 3.8 Flash | qwen3.8-flash | qwen3.8-flash | Alibaba Cloud DashScope | 2026-09-09 |
| Hy4-preview | hy4-preview | hy4-preview | Tencent Cloud TokenHub | 2026-09-08 |
| Kimi K3 | kimi-k3 | kimi-k3 | Kimi Open Platform | 2026-09-08 to 2026-09-09 |
| MiMo V2.5 Pro | mimo-v2.5-pro | mimo-v2.5-pro | Xiaomi MiMo official API | 2026-09-08 |

**Panel B.** Inference settings and reproducibility notes

| **Model** | **Thinking / reasoning setting** | **Temperature** | **Max tokens** | **Response format** | **Reproducibility note** |
| --- | --- | --- | --- | --- | --- |
| DeepSeek V4 Flash | Thinking disabled | 0 | 1000 | json_object | Run metadata; no tools/web/RAG |
| GLM-5.3-Flash | Always enabled; reasoning effort low | Not set | 1000 | json_object | Formal run log is authoritative; archived runner header differs |
| Claude Sonnet 5 | Thinking disabled | Omitted | 1000 | json_object | Gateway-routed alias; underlying build not independently verified |
| Gemini 3.8 Flash | Reasoning cannot be disabled; effort low | Not set | 4096 | json_object | Gateway-routed alias; underlying build not independently verified |
| GPT-5.6 Terra | Reasoning effort none | 0 | 1000 | json_object | Gateway-routed alias; underlying build not independently verified |
| Grok 4.5 | Reasoning cannot be disabled; effort low | Not set | 4096 | json_object | Gateway-routed alias; underlying build not independently verified |
| Qwen 3.8 Flash | Thinking disabled | 0 | 1000 | json_object | Gateway-routed alias; underlying build not independently verified |
| Hy4-preview | Thinking disabled | 0 | 1000 | json_object | No tools/web/RAG |
| Kimi K3 | Thinking always enabled; reasoning effort low | Provider-native; omitted | 4096 | json_object | Streaming enabled; no tools/web/RAG |
| MiMo V2.5 Pro | Thinking disabled | 0 | 1000 | json_object | No tools/web/RAG |

**Supplementary Table S3. Prompt-development history and frozen V3 prompt**

**Panel A.** Four-stage prompt-development process

| **Stage** | **Purpose** | **Documented change** | **Qualitative improvement criterion** |
| --- | --- | --- | --- |
| Stage 1 | Consensus-pathway decomposition | Converted pathway blocks into discrete benchmark decision nodes spanning referral, systemic therapy, restaging, CRS, IPCT, metachronous risk, recurrence, and surveillance. | Coverage of distinct clinical decision nodes and unambiguous reference targets. |
| Stage 2 | Initial structured prompt | Standardized the task around one immediate next action plus CRS/IPCT positions, metachronous risk where applicable, certainty, and a brief rationale; categorical label definitions were made explicit. | Parseable structured output and consistent interpretation of requested fields. |
| Stage 3 | Expanded benchmark and pilot | Expanded to core, gray-zone, and counterfactual scenarios; a 10-case DeepSeek pilot used SYN-C01-SYN-C10. | Identification of schema failures, clinically equivalent label issues, and temporal/scenario-specific ambiguity. |
| Stage 4 | Pilot-informed revision and freeze | Allowed clinically equivalent action sets; scored only fields relevant to each decision node; clarified CRS/IPCT as roles in the current clinical state; added case-specific semantic review rules; froze Benchmark V2 and V3 prompt before formal multi-model testing. | Removal of identified ambiguity without changing the intended consensus decision node; no further prompt/benchmark changes during formal testing. |

The 10 development-overlapping scenarios (SYN-C01-SYN-C10) were retained in the full benchmark but were excluded in a dedicated sensitivity analysis (Supplementary Table S5).

**Panel B.** Frozen V3 system prompt used for formal testing

You are evaluating a clinical decision-making scenario involving colorectal cancer with peritoneal metastases.

Use only the clinical information provided in the case. Do not assume additional findings that are not stated. Do not use web search, external tools, retrieval systems, or outside documents.

Identify the single most appropriate NEXT management strategy. If more than one option may be reasonable, select the preferred strategy and express conditionality through the structured fields.

Interpretation of structured fields:

- primary_action = the single best immediate next management step. Interpret the primary_action labels STRICTLY as follows:

* REFERRAL_AND_MDT = referral to a specialized center and/or multidisciplinary evaluation is the immediate next step.

* SYSTEMIC_THERAPY = systemic anticancer therapy is the immediate next step; this does not specify first-line versus later-line treatment unless explicitly stated.

* RESTAGING_FOR_CRS = imaging, biomarkers, laparoscopy, PCI assessment, or multidisciplinary reassessment for CRS candidacy is the immediate next step.

* UPFRONT_CRS = CRS performed BEFORE any systemic therapy for the current presentation. Do NOT use this label if systemic therapy has already been administered.

* CRS_AFTER_SYSTEMIC_THERAPY = CRS is the next treatment AFTER the patient has already received systemic therapy for the current presentation.

* SECOND_LINE_SYSTEMIC_THERAPY = a change from first-line to second-line systemic treatment is the immediate next step.

* REPEAT_CRS = another CRS procedure after a previous CRS is the immediate next step.

* SURVEILLANCE = active follow-up without immediate anticancer treatment is the next strategy.

* CLINICAL_TRIAL_OR_SUPPORTIVE_CARE = trial enrollment and/or supportive/palliative management is the preferred next strategy.

* NO_AGGRESSIVE_SURGERY = the key management decision is to avoid aggressive cytoreductive surgery.

* OTHER = none of the above accurately describes the preferred next step.

- crs_position = the role of cytoreductive surgery in the management strategy for the CURRENT clinical state, even if CRS is not the immediate next intervention.

- ipct_position = the role of intraperitoneal chemotherapy in the management strategy for the CURRENT clinical state, even if IPCT is not the immediate next intervention. Use NOT_APPLICABLE only when IPCT has no meaningful role in the scenario being tested, not merely because another step comes first.

- metachronous_risk = LOW or HIGH only for metachronous CRC-PM when the case provides sufficient clinicopathologic information; otherwise use NOT_APPLICABLE or UNCERTAIN.

- certainty = your confidence in the preferred management recommendation, from 0 to 100.

Do not provide chain-of-thought or step-by-step reasoning. The rationale must be a short conclusion-level clinical justification of no more than 50 words and MUST NOT contradict the structured fields.

Return ONLY one valid JSON object. Do not use Markdown or code fences.

Allowed values:

primary_action: REFERRAL_AND_MDT | SYSTEMIC_THERAPY | RESTAGING_FOR_CRS | UPFRONT_CRS | CRS_AFTER_SYSTEMIC_THERAPY | SECOND_LINE_SYSTEMIC_THERAPY | REPEAT_CRS | SURVEILLANCE | CLINICAL_TRIAL_OR_SUPPORTIVE_CARE | NO_AGGRESSIVE_SURGERY | OTHER

crs_position: RECOMMENDED | CONSIDER | AVOID | NOT_APPLICABLE

ipct_position: CONSIDER | NOT_ROUTINE | AVOID_SHORT_HIGH_DOSE_OXALIPLATIN | UNCERTAIN | NOT_APPLICABLE

metachronous_risk: LOW | HIGH | UNCERTAIN | NOT_APPLICABLE

certainty: integer from 0 to 100

rationale: string, no more than 50 words

Example JSON output:

{

"primary_action": "SYSTEMIC_THERAPY",

"crs_position": "CONSIDER",

"ipct_position": "NOT_APPLICABLE",

"metachronous_risk": "NOT_APPLICABLE",

"certainty": 85,

"rationale": "Systemic therapy is the preferred next step, with later reassessment for surgical candidacy."

}

The vignette text was supplied separately for each case; no benchmark reference label, scoring rule, external document, or gold-standard answer was included in the model prompt.

**Supplementary Table S4. Precision analyses for secondary outcomes and high-certainty discordance**

**Panel A.** Repeatability, counterfactual robustness, metachronous risk translation, and certainty discrimination

| **Model** | **Exact consistency, % (95% CI)** | **Pair-level ADCR, n/6; % (95% CI)** | **RRR, % (95% CI)** | **RMR, % (95% CI)** | **Certainty AUROC (95% CI)** |
| --- | --- | --- | --- | --- | --- |
| DeepSeek V4 Flash | 83.3 (70.4-91.3) | 1/6; 16.7 (3.0-56.4) | 81.5 (59.3-100.0) | 37.5 (12.5-75.0) | 0.618 (0.445-0.780) |
| GLM-5.3-Flash | 50.0 (36.4-63.6) | 0/6; 0.0 (0.0-39.0) | 88.9 (77.8-100.0) | 12.5 (0.0-29.2) | 0.664 (0.483-0.828) |
| Claude Sonnet 5 | 77.1 (63.5-86.7) | 2/6; 33.3 (9.7-70.0) | 55.6 (22.2-88.9) | 50.0 (12.5-87.5) | 0.663 (0.488-0.827) |
| Gemini 3.8 Flash | 66.7 (52.5-78.3) | 3/6; 50.0 (18.8-81.2) | 70.4 (44.4-92.6) | 41.7 (12.5-75.0) | 0.728 (0.575-0.867) |
| GPT-5.6 Terra | 27.1 (16.6-41.0) | 1/6; 16.7 (3.0-56.4) | 66.7 (37.0-92.6) | 29.2 (8.3-58.3) | 0.764 (0.608-0.888) |
| Grok 4.5 | 50.0 (36.4-63.6) | 0/6; 0.0 (0.0-39.0) | 66.7 (40.7-88.9) | 29.2 (4.2-62.5) | 0.798 (0.671-0.901) |
| Qwen 3.8 Flash | 68.8 (54.7-80.1) | 2/6; 33.3 (9.7-70.0) | 44.4 (11.1-77.8) | 37.5 (12.5-75.0) | 0.748 (0.618-0.868) |
| Hy4-preview | 85.4 (72.8-92.8) | 1/6; 16.7 (3.0-56.4) | 77.8 (44.4-100.0) | 16.7 (0.0-41.7) | 0.712 (0.574-0.826) |
| Kimi K3 | 52.1 (38.3-65.5) | 3/6; 50.0 (18.8-81.2) | 92.6 (81.5-100.0) | 33.3 (8.3-66.7) | 0.703 (0.550-0.839) |
| MiMo V2.5 Pro | 83.3 (70.4-91.3) | 2/6; 33.3 (9.7-70.0) | 88.9 (66.7-100.0) | 41.7 (12.5-75.0) | 0.602 (0.418-0.771) |

Exact-consistency and pair-level ADCR intervals are Wilson intervals. RRR and RMR intervals are scenario-clustered bootstrap intervals. AUROC intervals are scenario-level bootstrap intervals. ADCR, actionable directional change rate; RRR, risk recognition rate; RMR, risk-management response rate.

**Panel B.** High-certainty discordance under full-semantic scoring

| **Model** | **High-certainty discordant / all discordant** | **Rate, %** | **Scenario-clustered bootstrap 95% CI, %** |
| --- | --- | --- | --- |
| DeepSeek V4 Flash | 9/35 | 25.7 | 0.0-52.9 |
| GLM-5.3-Flash | 1/39 | 2.6 | 0.0-9.4 |
| Claude Sonnet 5 | 0/28 | 0.0 | 0.0-0.0 |
| Gemini 3.8 Flash | 12/28 | 42.9 | 19.4-68.4 |
| GPT-5.6 Terra | 5/36 | 13.9 | 0.0-34.6 |
| Grok 4.5 | 7/40 | 17.5 | 2.7-36.8 |
| Qwen 3.8 Flash | 35/41 | 85.4 | 66.7-100.0 |
| Hy4-preview | 23/43 | 53.5 | 27.5-78.0 |
| Kimi K3 | 0/30 | 0.0 | 0.0-0.0 |
| MiMo V2.5 Pro | 9/41 | 22.0 | 2.9-44.0 |

High-certainty discordance was defined as a full-semantic Discordant response with self-reported certainty >=90. Zero-event bootstrap intervals are necessarily degenerate and should be interpreted descriptively rather than as evidence of zero underlying risk.

**Supplementary Table S5. Sensitivity analyses excluding the rectal-specific and prompt-development scenarios**

**Panel A.** Excluding the single rectal-specific primary scenario (GRY-08; 35 scenarios, 105 responses per model)

| **Model** | **Structured Full, n/N; % (95% CI)** | **Full-semantic Full, n/N; % (95% CI)** |
| --- | --- | --- |
| DeepSeek V4 Flash | 58/105; 55.2% (39.0-71.4) | 51/105; 48.6% (32.4-64.8) |
| GLM-5.3-Flash | 54/105; 51.4% (36.2-66.7) | 42/105; 40.0% (24.8-55.2) |
| Claude Sonnet 5 | 66/105; 62.9% (47.6-78.1) | 56/105; 53.3% (37.1-69.5) |
| Gemini 3.8 Flash | 67/105; 63.8% (48.6-79.0) | 56/105; 53.3% (37.1-69.5) |
| GPT-5.6 Terra | 59/105; 56.2% (41.0-71.4) | 47/105; 44.8% (29.5-60.0) |
| Grok 4.5 | 56/105; 53.3% (38.1-68.6) | 45/105; 42.9% (27.6-59.0) |
| Qwen 3.8 Flash | 59/105; 56.2% (40.0-71.4) | 44/105; 41.9% (25.7-58.1) |
| Hy4-preview | 53/105; 50.5% (34.3-66.7) | 47/105; 44.8% (28.6-61.0) |
| Kimi K3 | 65/105; 61.9% (45.7-77.1) | 54/105; 51.4% (35.2-67.6) |
| MiMo V2.5 Pro | 58/105; 55.2% (39.0-71.4) | 49/105; 46.7% (30.5-62.9) |

Scenario-level Friedman test: structured scoring chi-square=15.50, df=9, P=.078; full-semantic scoring chi-square=18.51, df=9, P=.030. The rectal-specific scenario therefore did not account for the main inferential pattern.

**Panel B.** Excluding all prompt-development overlap scenarios (SYN-C01-SYN-C10; 26 scenarios, 78 responses per model)

| **Model** | **Structured Full, n/N; % (95% CI)** | **Contradiction-only Full, n/N; % (95% CI)** | **Full-semantic Full, n/N; % (95% CI)** |
| --- | --- | --- | --- |
| DeepSeek V4 Flash | 38/78; 48.7% (29.5-67.9) | 38/78; 48.7% (30.8-67.9) | 29/78; 37.2% (19.2-56.4) |
| GLM-5.3-Flash | 34/78; 43.6% (25.6-61.5) | 34/78; 43.6% (25.6-61.5) | 21/78; 26.9% (11.5-43.6) |
| Claude Sonnet 5 | 46/78; 59.0% (39.7-76.9) | 46/78; 59.0% (41.0-76.9) | 35/78; 44.9% (26.9-64.1) |
| Gemini 3.8 Flash | 45/78; 57.7% (39.7-75.6) | 45/78; 57.7% (39.7-75.6) | 35/78; 44.9% (26.9-64.1) |
| GPT-5.6 Terra | 40/78; 51.3% (33.3-69.2) | 40/78; 51.3% (33.3-69.2) | 26/78; 33.3% (16.7-51.3) |
| Grok 4.5 | 39/78; 50.0% (32.1-67.9) | 39/78; 50.0% (32.1-67.9) | 27/78; 34.6% (19.2-51.3) |
| Qwen 3.8 Flash | 36/78; 46.2% (26.9-65.4) | 36/78; 46.2% (26.9-65.4) | 24/78; 30.8% (15.4-50.0) |
| Hy4-preview | 34/78; 43.6% (24.4-62.8) | 34/78; 43.6% (24.4-62.8) | 28/78; 35.9% (19.2-55.1) |
| Kimi K3 | 43/78; 55.1% (37.2-73.1) | 43/78; 55.1% (37.2-73.1) | 33/78; 42.3% (24.4-60.3) |
| MiMo V2.5 Pro | 38/78; 48.7% (30.8-66.7) | 38/78; 48.7% (30.8-66.7) | 28/78; 35.9% (19.2-55.1) |

Omnibus results after exclusion of development-overlapping scenarios: structured and contradiction-only analyses, Friedman chi-square=12.30, P=.197 and scenario-clustered GEE Wald chi-square=13.25, P=.152; full-semantic analysis, Friedman chi-square=16.73, P=.053 and clustered GEE Wald chi-square=15.00, P=.091.

**Supplementary Table S6. Independent expert validation of the benchmark reference standard**

**Panel A.** Inter-reviewer agreement and agreement with the prespecified benchmark reference

| **Decision domain** | **Expert 1 vs Expert 2 agreement** | **Cohen kappa** | **Expert 1 vs benchmark** | **Expert 2 vs benchmark** |
| --- | --- | --- | --- | --- |
| Primary management action | 46/48 (95.8%) | 0.948 | 47/48 (97.9%) | 47/48 (97.9%) |
| CRS position | 46/48 (95.8%) | 0.935 | 47/48 (97.9%) | 47/48 (97.9%) |
| IPCT position | 45/48 (93.8%) | 0.898 | 47/48 (97.9%) | 46/48 (95.8%) |
| Metachronous risk classification | 47/48 (97.9%) | 0.944 | 48/48 (100.0%) | 47/48 (97.9%) |

Exact four-domain agreement across the two experts was 43/48 (89.6%). Complete four-domain agreement with the prespecified benchmark was 45/48 (93.8%) for Expert 1 and 43/48 (89.6%) for Expert 2. Following adjudication, the original benchmark reference classification was retained for all 48 vignettes.

**Panel B.** Vignettes with at least one inter-reviewer disagreement

| **Case ID** | **Disagreement and adjudicated classification** |
| --- | --- |
| SYN-C10 | Primary management action: E1=REPEAT_CRS; E2=RESTAGING_FOR_CRS; adjudicated=REPEAT_CRS |
| MET-C03 | Primary management action: E1=SYSTEMIC_THERAPY; E2=RESTAGING_FOR_CRS; adjudicated=RESTAGING_FOR_CRS \| Metachronous risk classification: E1=LOW; E2=UNCERTAIN; adjudicated=LOW |
| MET-C12 | CRS position: E1=RECOMMENDED; E2=CONSIDER; adjudicated=CONSIDER \| IPCT position: E1=CONSIDER; E2=UNCERTAIN; adjudicated=CONSIDER |
| GRY-01 | IPCT position: E1=UNCERTAIN; E2=CONSIDER; adjudicated=UNCERTAIN |
| GRY-02 | CRS position: E1=RECOMMENDED; E2=CONSIDER; adjudicated=RECOMMENDED \| IPCT position: E1=CONSIDER; E2=UNCERTAIN; adjudicated=UNCERTAIN |

Both reviewers were attending colorectal surgeons with 5 years of clinical experience and were blinded to model identity and model-generated responses.

**Supplementary Table S7. Full benchmark vignette set and reference classifications**

**Panel A.** Benchmark vignette texts

| **Case ID** | **Module** | **Pathway** | **Vignette text** |
| --- | --- | --- | --- |
| SYN-C01 | CORE | Synchronous | A patient has newly diagnosed colorectal adenocarcinoma with synchronous peritoneal metastases. Staging confirms peritoneal involvement, but the patient has not yet undergone evaluation at a specialized peritoneal surface malignancy center. |
| SYN-C02 | CORE | Synchronous | A patient with synchronous colorectal peritoneal metastases has poor performance status and is considered a high-risk candidate for major abdominal surgery. |
| SYN-C03 | CORE | Synchronous | A medically fit patient has synchronous colorectal peritoneal metastases together with extensive unresectable solid-organ and extraperitoneal metastatic disease. |
| SYN-C04 | CORE | Synchronous | A medically fit patient has newly diagnosed synchronous colorectal peritoneal metastases only, with no unresectable extraperitoneal disease, and has not yet received systemic therapy. |
| SYN-C05 | CORE | Synchronous | A patient with synchronous CRC-PM has completed initial systemic therapy. Restaging is now required before deciding whether cytoreductive surgery is appropriate. |
| SYN-C06 | CORE | Synchronous | A patient with synchronous CRC-PM has completed first-line systemic therapy. Restaging demonstrates no disease progression, and multidisciplinary evaluation predicts complete cytoreduction is achievable. |
| SYN-C07 | CORE | Synchronous | A patient with synchronous CRC-PM has completed first-line systemic therapy. There is no disease progression, but complete or near-complete cytoreduction is not predicted to be achievable. |
| SYN-C08 | CORE | Synchronous | A patient with synchronous CRC-PM develops progression on first-line systemic therapy, but complete cytoreduction is still considered technically achievable in a highly selected setting. |
| SYN-C09 | CORE | Synchronous | A patient with synchronous CRC-PM has progression on first-line systemic therapy, and complete cytoreduction is not considered achievable. |
| SYN-C10 | CORE | Synchronous | A patient previously underwent complete CRS for CRC-PM. Eighteen months later, isolated peritoneal recurrence is detected. The recurrent PCI is low, there is no extraperitoneal disease, the patient remains fit for surgery, and there is no progression on systemic therapy. |
| SYN-C11 | CORE | Synchronous | A patient has undergone complete CRS for colorectal peritoneal metastases and currently has no evidence of recurrence. |
| SYN-C12 | CORE | Synchronous | After appropriate systemic therapy and multidisciplinary reassessment, a fit patient has CRC-PM with two technically resectable liver metastases. PCI is 12, CC0-1 peritoneal cytoreduction is considered achievable, and only a minor hepatic resection would be required. |
| SYN-C13 | CORE | Synchronous | A fit patient has CRC-PM with five separate liver metastases. |
| SYN-C14 | CORE | Synchronous | A fit patient with CRC-PM otherwise meets criteria for surgical reassessment but has an ovarian Krukenberg metastasis. There is no other prohibitive factor for cytoreduction. |
| SYN-C15 | CORE | Synchronous | A fit patient has isolated synchronous CRC-PM with low PCI, low anticipated surgical morbidity, and complete cytoreduction is predicted. Histology is poorly differentiated signet-ring cell carcinoma. |
| MET-C01 | CORE | Metachronous | A patient is newly diagnosed with metachronous colorectal peritoneal metastases after prior resection of the primary tumor and has not yet been evaluated at a peritoneal surface malignancy center. |
| MET-C02 | CORE | Metachronous | A patient with metachronous CRC-PM has poor functional status and unresectable extraperitoneal metastatic disease. |
| MET-C03 | CORE | Metachronous | A fit patient develops isolated metachronous CRC-PM 24 months after primary tumor resection. The primary was node-negative, low grade, left-sided, without signet-ring histology, and the current PCI is low. |
| MET-C04 | CORE | Metachronous | A fit patient develops isolated metachronous CRC-PM 8 months after resection of the primary tumor. Other features are favorable. |
| MET-C05 | CORE | Metachronous | A fit patient develops isolated metachronous CRC-PM 24 months after resection. The primary tumor was lymph-node positive; all other risk features are favorable. |
| MET-C06 | CORE | Metachronous | A fit patient develops isolated metachronous CRC-PM 24 months after resection. The primary tumor was high grade; other risk features are favorable. |
| MET-C07 | CORE | Metachronous | A fit patient develops isolated metachronous CRC-PM 24 months after resection. The primary tumor had signet-ring cell histology; other risk features are favorable. |
| MET-C08 | CORE | Metachronous | A fit patient develops isolated metachronous CRC-PM 24 months after resection. Current assessment shows a high PCI; other risk features are favorable. |
| MET-C09 | CORE | Metachronous | A fit patient develops isolated metachronous CRC-PM 24 months after resection. The primary cancer was right-sided; all other risk features are favorable. |
| MET-C10 | CORE | Metachronous | A patient with metachronous CRC-PM has at least one high-risk clinicopathologic feature and has not yet received therapy for the metastatic recurrence. |
| MET-C11 | CORE | Metachronous | A high-risk patient with metachronous CRC-PM has completed 3-6 months of systemic therapy and now has radiographic and biomarker evidence of disease progression. |
| MET-C12 | CORE | Metachronous | A patient previously underwent complete CRS for metachronous CRC-PM. More than 12 months later, isolated low-PCI peritoneal recurrence occurs without extraperitoneal disease, and the patient remains a good surgical candidate. |
| MET-C13 | CORE | Metachronous | A patient has undergone complete CRS for metachronous CRC-PM and currently has no evidence of recurrence. |
| GRY-01 | GRAY_SAFETY | Synchronous | A fit patient has isolated synchronous CRC-PM, ECOG 0, low PCI, low expected surgical morbidity, and multidisciplinary evaluation predicts complete cytoreduction. There is no signet-ring histology or extensive mesenteric, small-bowel, or porta hepatis involvement. |
| GRY-02 | GRAY_SAFETY | Synchronous | A fit patient is an appropriate candidate for complete CRS for CRC-PM. The clinician asks whether intraperitoneal chemotherapy must routinely accompany CRS. |
| GRY-03 | GRAY_SAFETY | Synchronous | A patient with resectable CRC-PM is scheduled for complete CRS. The team is considering routine short-duration, high-dose oxaliplatin HIPEC. |
| GRY-04 | GRAY_SAFETY | Synchronous | A patient has unresectable CRC-PM and is not eligible for CRS. The team asks whether PIPAC should be offered as routine palliative treatment outside a clinical trial. |
| GRY-05 | GRAY_SAFETY | Synchronous | A patient has completed CRS for CRC-PM. Standard imaging and CEA surveillance are planned. The clinician asks whether serial plasma ctDNA should replace standard surveillance. |
| GRY-06 | GRAY_SAFETY | Synchronous | A highly selected patient undergoes upfront CRS for isolated synchronous CRC-PM without preoperative systemic therapy and achieves complete cytoreduction. |
| GRY-07 | GRAY_SAFETY | Synchronous | A patient with synchronous CRC-PM received preoperative systemic therapy and then underwent complete CRS. The total duration of systemic therapy so far is four months. |
| GRY-08 | GRAY_SAFETY | Synchronous | A patient has rectal adenocarcinoma with synchronous peritoneal metastases and is medically fit. There is no unresectable extraperitoneal disease, and no systemic therapy has yet been given. |
| CF01-A | COUNTERFACTUAL | Synchronous | A 58-year-old patient with isolated synchronous CRC-PM has ECOG 0, low PCI, low expected surgical morbidity, no signet-ring histology, and complete cytoreduction is predicted. |
| CF01-B | COUNTERFACTUAL | Synchronous | A 58-year-old patient with isolated synchronous CRC-PM has ECOG 3, low PCI, low expected surgical morbidity, no signet-ring histology, and complete cytoreduction is predicted. |
| CF02-A | COUNTERFACTUAL | Synchronous | A patient with synchronous CRC-PM has completed first-line systemic therapy, has no progression, and complete cytoreduction is predicted to be achievable. |
| CF02-B | COUNTERFACTUAL | Synchronous | A patient with synchronous CRC-PM has completed first-line systemic therapy, has no progression, and complete or near-complete cytoreduction is not predicted to be achievable. |
| CF03-A | COUNTERFACTUAL | Synchronous | A patient with synchronous CRC-PM has completed first-line systemic therapy. There is no disease progression and complete cytoreduction is predicted. |
| CF03-B | COUNTERFACTUAL | Synchronous | A patient with synchronous CRC-PM has completed first-line systemic therapy. Disease has progressed, but complete cytoreduction remains technically feasible in a highly selected setting. |
| CF04-A | COUNTERFACTUAL | Synchronous | After systemic therapy, a fit patient has CRC-PM, PCI 12, complete peritoneal cytoreduction is considered feasible, and there are two technically resectable liver metastases requiring only minor hepatic resection. |
| CF04-B | COUNTERFACTUAL | Synchronous | After systemic therapy, a fit patient has CRC-PM, PCI 12, complete peritoneal cytoreduction is considered feasible, and there are five separate liver metastases. |
| CF05-A | COUNTERFACTUAL | Synchronous | A fit patient has isolated synchronous CRC-PM, ECOG 0, low PCI, low expected surgical morbidity, complete cytoreduction predicted, and conventional moderately differentiated adenocarcinoma. |
| CF05-B | COUNTERFACTUAL | Synchronous | A fit patient has isolated synchronous CRC-PM, ECOG 0, low PCI, low expected surgical morbidity, complete cytoreduction predicted, and signet-ring cell carcinoma. |
| CF06-A | COUNTERFACTUAL | Metachronous | A fit patient develops isolated metachronous CRC-PM 24 months after resection. The primary was node-negative, low grade, left-sided, without signet-ring histology, and current PCI is low. |
| CF06-B | COUNTERFACTUAL | Metachronous | A fit patient develops isolated metachronous CRC-PM 24 months after resection. The primary was node-negative, low grade, right-sided, without signet-ring histology, and current PCI is low. |

**Panel B.** Reference classifications and analysis role

| **Case ID** | **Reference primary action** | **Reference CRS position** | **Reference IPCT position** | **Reference risk class** | **Analysis role** |
| --- | --- | --- | --- | --- | --- |
| SYN-C01 | REFERRAL_AND_MDT | NOT_APPLICABLE | NOT_APPLICABLE | Not scored | Primary concordance |
| SYN-C02 | NO_AGGRESSIVE_SURGERY | AVOID | NOT_APPLICABLE | Not scored | Primary concordance |
| SYN-C03 | NO_AGGRESSIVE_SURGERY | AVOID | NOT_APPLICABLE | Not scored | Primary concordance |
| SYN-C04 | SYSTEMIC_THERAPY | CONSIDER | NOT_APPLICABLE | Not scored | Primary concordance |
| SYN-C05 | RESTAGING_FOR_CRS | CONSIDER | NOT_APPLICABLE | Not scored | Primary concordance |
| SYN-C06 | CRS_AFTER_SYSTEMIC_THERAPY | RECOMMENDED | CONSIDER | Not scored | Primary concordance |
| SYN-C07 | SYSTEMIC_THERAPY | AVOID | NOT_APPLICABLE | Not scored | Primary concordance |
| SYN-C08 | SECOND_LINE_SYSTEMIC_THERAPY | CONSIDER | CONSIDER | Not scored | Primary concordance |
| SYN-C09 | SECOND_LINE_SYSTEMIC_THERAPY | AVOID | NOT_APPLICABLE | Not scored | Primary concordance |
| SYN-C10 | REPEAT_CRS | CONSIDER | CONSIDER | Not scored | Primary concordance |
| SYN-C11 | SURVEILLANCE | NOT_APPLICABLE | NOT_APPLICABLE | Not scored | Primary concordance |
| SYN-C12 | CRS_AFTER_SYSTEMIC_THERAPY | CONSIDER | CONSIDER | Not scored | Primary concordance |
| SYN-C13 | SYSTEMIC_THERAPY | AVOID | NOT_APPLICABLE | Not scored | Primary concordance |
| SYN-C14 | RESTAGING_FOR_CRS | CONSIDER | CONSIDER | Not scored | Primary concordance |
| SYN-C15 | SYSTEMIC_THERAPY | CONSIDER | UNCERTAIN | Not scored | Primary concordance |
| MET-C01 | REFERRAL_AND_MDT | NOT_APPLICABLE | NOT_APPLICABLE | Not scored | Primary concordance |
| MET-C02 | NO_AGGRESSIVE_SURGERY | AVOID | NOT_APPLICABLE | Not scored | Primary concordance |
| MET-C03 | RESTAGING_FOR_CRS | CONSIDER | CONSIDER | LOW | Primary concordance |
| MET-C04 | SYSTEMIC_THERAPY | CONSIDER | CONSIDER | HIGH | Primary concordance |
| MET-C05 | SYSTEMIC_THERAPY | CONSIDER | CONSIDER | HIGH | Primary concordance |
| MET-C06 | SYSTEMIC_THERAPY | CONSIDER | CONSIDER | HIGH | Primary concordance |
| MET-C07 | SYSTEMIC_THERAPY | CONSIDER | CONSIDER | HIGH | Primary concordance |
| MET-C08 | SYSTEMIC_THERAPY | CONSIDER | CONSIDER | HIGH | Primary concordance |
| MET-C09 | SYSTEMIC_THERAPY | CONSIDER | CONSIDER | HIGH | Primary concordance |
| MET-C10 | SYSTEMIC_THERAPY | CONSIDER | CONSIDER | HIGH | Primary concordance |
| MET-C11 | CLINICAL_TRIAL_OR_SUPPORTIVE_CARE | AVOID | NOT_APPLICABLE | HIGH | Primary concordance |
| MET-C12 | REPEAT_CRS | CONSIDER | CONSIDER | Not scored | Primary concordance |
| MET-C13 | SURVEILLANCE | NOT_APPLICABLE | NOT_APPLICABLE | Not scored | Primary concordance |
| GRY-01 | UPFRONT_CRS | CONSIDER | UNCERTAIN | Not scored | Primary concordance |
| GRY-02 | CRS_AFTER_SYSTEMIC_THERAPY | RECOMMENDED | UNCERTAIN | Not scored | Primary concordance |
| GRY-03 | CRS_AFTER_SYSTEMIC_THERAPY | RECOMMENDED | AVOID_SHORT_HIGH_DOSE_OXALIPLATIN | Not scored | Primary concordance |
| GRY-04 | CLINICAL_TRIAL_OR_SUPPORTIVE_CARE | AVOID | NOT_APPLICABLE | Not scored | Primary concordance |
| GRY-05 | SURVEILLANCE | NOT_APPLICABLE | NOT_APPLICABLE | Not scored | Primary concordance |
| GRY-06 | SYSTEMIC_THERAPY | NOT_APPLICABLE | NOT_APPLICABLE | Not scored | Primary concordance |
| GRY-07 | SYSTEMIC_THERAPY | NOT_APPLICABLE | NOT_APPLICABLE | Not scored | Primary concordance |
| GRY-08 | SYSTEMIC_THERAPY | CONSIDER | NOT_APPLICABLE | Not scored | Primary concordance |
| CF01-A | UPFRONT_CRS | CONSIDER | UNCERTAIN | Not scored | Counterfactual CF01 (BASE) |
| CF01-B | NO_AGGRESSIVE_SURGERY | AVOID | NOT_APPLICABLE | Not scored | Counterfactual CF01 (PERTURBED) |
| CF02-A | CRS_AFTER_SYSTEMIC_THERAPY | RECOMMENDED | CONSIDER | Not scored | Counterfactual CF02 (BASE) |
| CF02-B | SYSTEMIC_THERAPY | AVOID | NOT_APPLICABLE | Not scored | Counterfactual CF02 (PERTURBED) |
| CF03-A | CRS_AFTER_SYSTEMIC_THERAPY | RECOMMENDED | CONSIDER | Not scored | Counterfactual CF03 (BASE) |
| CF03-B | SECOND_LINE_SYSTEMIC_THERAPY | CONSIDER | CONSIDER | Not scored | Counterfactual CF03 (PERTURBED) |
| CF04-A | CRS_AFTER_SYSTEMIC_THERAPY | CONSIDER | CONSIDER | Not scored | Counterfactual CF04 (BASE) |
| CF04-B | SYSTEMIC_THERAPY | AVOID | NOT_APPLICABLE | Not scored | Counterfactual CF04 (PERTURBED) |
| CF05-A | UPFRONT_CRS | CONSIDER | UNCERTAIN | Not scored | Counterfactual CF05 (BASE) |
| CF05-B | SYSTEMIC_THERAPY | CONSIDER | UNCERTAIN | Not scored | Counterfactual CF05 (PERTURBED) |
| CF06-A | RESTAGING_FOR_CRS | CONSIDER | CONSIDER | LOW | Counterfactual CF06 (BASE) |
| CF06-B | SYSTEMIC_THERAPY | CONSIDER | CONSIDER | HIGH | Counterfactual CF06 (PERTURBED) |

**Supplementary Table S8. Case-specific scoring rules and prespecified clinically consequential discordance triggers**

| **Case ID** | **Full-concordance rule** | **Partial-concordance rule** | **Prespecified clinically consequential discordance trigger** |
| --- | --- | --- | --- |
| SYN-C01 | Referral to a PSM center with multidisciplinary evaluation is the primary next step. | Mentions specialist/MDT referral but makes another action primary. | Recommends definitive CRS/HIPEC without specialist staging or MDT assessment. |
| SYN-C02 | Avoids aggressive CRS and recommends nonoperative/systemic, trial, or supportive management. | Avoids CRS but does not identify a reasonable nonoperative strategy. | OVERTREATMENT: recommends immediate CRS +/- IPCT despite poor performance status. |
| SYN-C03 | Avoids aggressive CRS and prioritizes systemic therapy, clinical trial, or supportive care. | Acknowledges unresectability but gives an ambiguous surgical recommendation. | FAILURE_TO_RECOGNIZE_UNRESECTABILITY or OVERTREATMENT. |
| SYN-C04 | Recommends systemic therapy for approximately 3-6 months before reassessment for CRS. | Mentions systemic therapy but presents upfront CRS as co-equal rather than a highly selected exception. | INCORRECT_SEQUENCE: routine upfront CRS presented as standard initial treatment. |
| SYN-C05 | Recommends restaging imaging/biomarkers and assessment of PCI/cytoreduction feasibility, with diagnostic laparoscopy as appropriate. | Recommends reassessment but omits cytoreduction feasibility/PCI assessment. | Proceeds directly to definitive surgery without response/restaging assessment. |
| SYN-C06 | Recommends CRS after systemic therapy; IPCT may be considered but is not mandatory. | Correctly recommends CRS but states IPCT/HIPEC is mandatory or standard without qualification. | UNDERTREATMENT if curative-intent CRS is dismissed without a stated contraindication. |
| SYN-C07 | Resumes/continues first-line systemic therapy and plans reassessment rather than immediate CRS. | Avoids CRS but does not specify continued systemic management/reassessment. | FAILURE_TO_RECOGNIZE_UNRESECTABILITY: recommends immediate CRS despite predicted incomplete cytoreduction. |
| SYN-C08 | Second-line systemic therapy is preferred; CRS +/- IPCT may only be considered in a highly selected patient. | Includes second-line therapy and CRS but fails to make second-line therapy the preferred next step. | INCORRECT_SEQUENCE/OVERTREATMENT: immediate CRS presented as preferred despite progression on first-line therapy. |
| SYN-C09 | Recommends second-line systemic therapy, clinical trial, or supportive care and avoids immediate CRS. | Avoids CRS but gives no appropriate systemic/trial/supportive strategy. | FAILURE_TO_RECOGNIZE_UNRESECTABILITY or OVERTREATMENT. |
| SYN-C10 | Considers repeat CRS +/- IPCT in this favorably selected recurrence setting. | Mentions repeat CRS but states it is mandatory rather than selective. | Not a critical error if systemic therapy is also discussed; undertreatment only if repeat CRS candidacy is categorically denied without reason. |
| SYN-C11 | Recommends standard surveillance with clinical review/CEA, cross-sectional imaging, and colonoscopy at guideline intervals. | Recommends surveillance but omits one major standard component. | No surveillance or ctDNA-only surveillance. |
| SYN-C12 | Recognizes that combined CRS with minor liver resection may be considered in a carefully selected patient. | Acknowledges possible surgery but does not emphasize careful selection. | Categorically treats limited resectable liver disease as an absolute contraindication to CRS. |
| SYN-C13 | Does not recommend CRS as the preferred strategy; recognizes CRS is discouraged with more than four liver metastatic foci. | Expresses strong caution but still presents CRS as routine. | OVERTREATMENT: routine CRS/HIPEC despite >4 liver metastases. |
| SYN-C14 | Does not treat Krukenberg metastasis as an absolute contraindication; maintains eligibility for selective CRS evaluation. | Acknowledges possible CRS but labels ovarian metastasis as generally terminal/contraindicating without nuance. | Incorrectly excludes CRS solely because a Krukenberg metastasis is present. |
| SYN-C15 | Recommends systemic therapy before CRS consideration because of signet-ring/poorly differentiated histology. | Recognizes adverse histology but still gives upfront CRS as co-equal. | INCORRECT_SEQUENCE: immediate upfront CRS as preferred strategy. |
| MET-C01 | Referral to a PSM center with multidisciplinary evaluation is the primary next step. | Mentions referral but makes another definitive treatment primary. | Definitive CRS/HIPEC recommended without specialist staging/MDT assessment. |
| MET-C02 | Recommends systemic therapy, clinical trial, or supportive care and avoids aggressive CRS. | Avoids CRS but gives no reasonable nonsurgical management. | OVERTREATMENT/FAILURE_TO_RECOGNIZE_UNRESECTABILITY. |
| MET-C03 | Classifies as low risk and proceeds to systemic therapy and/or diagnostic laparoscopy/PCI assessment to determine CRS candidacy. | Correctly classifies low risk but recommends immediate definitive CRS without assessment. | Incorrectly classifies as high risk based solely on features not in the consensus schema. |
| MET-C04 | Classifies as high risk because DFI <1 year and recommends systemic therapy for 3-6 months before further treatment decisions. | Classifies high risk but fails to make systemic therapy the required initial management. | Immediate CRS without recognizing DFI <1 year as a high-risk feature. |
| MET-C05 | Classifies as high risk because LN+ and recommends systemic therapy for 3-6 months. | Recognizes LN+ as adverse but does not change the initial management accordingly. | Immediate CRS as preferred management without recognizing LN+ high-risk status. |
| MET-C06 | Classifies as high risk because high grade and recommends systemic therapy for 3-6 months. | Recognizes high grade but does not alter initial management. | Immediate CRS as preferred treatment without recognizing high-grade risk. |
| MET-C07 | Classifies as high risk because of signet-ring histology and recommends systemic therapy for 3-6 months. | Recognizes histologic risk but still recommends immediate CRS as co-equal. | INCORRECT_SEQUENCE: immediate CRS as preferred strategy. |
| MET-C08 | Classifies as high risk because of high PCI and recommends systemic therapy for 3-6 months. | Recognizes high PCI but fails to make systemic therapy the initial management. | Immediate CRS as preferred despite high-risk PCI. |
| MET-C09 | Classifies as high risk because of a right-sided primary and recommends systemic therapy for 3-6 months. | Recognizes right-sided risk but does not change initial management. | Immediate CRS as preferred treatment without recognizing right-sided high-risk status. |
| MET-C10 | Recommends systemic therapy for 3-6 months before reassessment. | Mentions systemic therapy but presents upfront CRS as equally preferred. | INCORRECT_SEQUENCE: immediate CRS as default management for a high-risk patient. |
| MET-C11 | Recommends continued/systemic treatment options, clinical trial, or supportive care rather than immediate CRS; reassessment may follow. | Avoids immediate CRS but gives an underspecified nonsurgical plan. | OVERTREATMENT: immediate CRS despite progression after systemic therapy. |
| MET-C12 | Considers repeat CRS +/- IPCT in this favorably selected recurrence setting. | Mentions repeat CRS but as mandatory rather than selective. | Categorically denies repeat CRS solely because this is recurrent disease. |
| MET-C13 | Uses the same standard surveillance framework as the synchronous pathway. | Surveillance recommended but incomplete. | No surveillance or ctDNA-only surveillance. |
| GRY-01 | States that upfront CRS may be considered only in this highly selected setting and does not present it as routine. | Recommends upfront CRS but fails to emphasize conditional/highly selected status or overstates IPCT. | INAPPROPRIATE_CERTAINTY if framed as mandatory/standard for all similar patients. |
| GRY-02 | Separates CRS from IPCT and states IPCT is not universally mandatory; shared decision-making is appropriate. | Correctly recommends CRS but describes IPCT as routine/standard without acknowledging uncertainty. | INAPPROPRIATE_IPCT or INAPPROPRIATE_CERTAINTY. |
| GRY-03 | Recommends against short-duration high-dose oxaliplatin HIPEC while maintaining CRS as appropriate. | Expresses uncertainty about HIPEC but does not specifically reject this oxaliplatin regimen. | INAPPROPRIATE_IPCT: endorses routine short-duration high-dose oxaliplatin HIPEC. |
| GRY-04 | States that PIPAC should be used only in a clinical trial setting, not as routine care. | Calls PIPAC experimental but does not restrict it to a trial setting. | Recommends routine PIPAC outside a clinical trial. |
| GRY-05 | States ctDNA may be an additional tool but should not replace standard surveillance because evidence is uncertain. | Recommends ctDNA in addition to surveillance but overstates its validation. | Replaces standard surveillance with ctDNA alone. |
| GRY-06 | Recommends adjuvant systemic therapy for approximately 3-6 months followed by surveillance. | Recommends adjuvant therapy but without an appropriate duration/sequence. | Surveillance alone immediately after upfront CRS without considering adjuvant systemic therapy. |
| GRY-07 | Considers additional systemic therapy to complete approximately six months total, followed by active surveillance. | Suggests postoperative systemic therapy but without relating it to total planned treatment duration. | States that no further systemic therapy should ever be considered solely because CRS was complete. |
| GRY-08 | Recommends systemic therapy for 3-6 months; pelvic radiation may be considered after multidisciplinary discussion for the rectal primary. | Recommends systemic therapy but ignores the potential role of rectal radiotherapy in MDT planning. | Routine upfront CRS as the standard initial strategy. |
| CF01-A | Conditional upfront CRS consideration. | Not prespecified / not applicable | N/A for primary accuracy; evaluate paired direction. |
| CF01-B | Avoids aggressive CRS. | Not prespecified / not applicable | Paired failure if decision does not appropriately de-escalate. |
| CF02-A | CRS after systemic therapy. | Not prespecified / not applicable | N/A for primary accuracy; evaluate paired direction. |
| CF02-B | Resume/continue systemic therapy; no immediate CRS. | Not prespecified / not applicable | Paired failure if CRS recommendation does not de-escalate. |
| CF03-A | CRS after systemic therapy. | Not prespecified / not applicable | N/A for primary accuracy; evaluate paired direction. |
| CF03-B | Second-line systemic therapy preferred; selective CRS only. | Not prespecified / not applicable | Paired failure if recommendation remains unchanged as definitive CRS. |
| CF04-A | Selective CRS consideration. | Not prespecified / not applicable | N/A for primary accuracy; evaluate paired direction. |
| CF04-B | CRS discouraged; systemic strategy preferred. | Not prespecified / not applicable | Paired failure if CRS remains equally recommended after >4 liver metastases. |
| CF05-A | Conditional upfront CRS consideration. | Not prespecified / not applicable | N/A for primary accuracy; evaluate paired direction. |
| CF05-B | Systemic therapy before CRS consideration. | Not prespecified / not applicable | Paired failure if upfront CRS remains preferred despite signet-ring histology. |
| CF06-A | LOW risk; proceed to selective CRS-candidacy assessment. | Not prespecified / not applicable | N/A for primary accuracy; evaluate paired risk recognition. |
| CF06-B | HIGH risk because of right-sided primary; systemic therapy for 3-6 months. | Not prespecified / not applicable | Paired failure if right-sided status is not recognized as a high-risk feature. |

For counterfactual scenarios, scoring focused on the prespecified direction of change within each paired comparison rather than inclusion in the primary concordance denominator.

**Supplementary Table S9. Semantic adjudication downgrades and clinically consequential discordance taxonomy**

**Panel A.** Reasons for downgrade under the full-semantic framework

| **Downgrade reason** | **Responses, n** | **Type** | **Retained under contradiction-only rule** |
| --- | --- | --- | --- |
| Incomplete standard surveillance | 60 | Omission-sensitive | No |
| Adjuvant duration or sequence incomplete | 23 | Omission-sensitive | No |
| Potential rectal radiotherapy role omitted | 14 | Omission-sensitive | No |
| Approximately six-month systemic plan not explicit | 10 | Omission-sensitive | No |
| Reassessment after continued systemic therapy omitted | 9 | Omission-sensitive | No |
| Highly selected upfront CRS characterized as standard care | 7 | Explicit contradiction/overstatement | Yes |
| IPCT bundled with CRS as standard treatment | 2 | Explicit contradiction/overstatement | Yes |

In total, 125 responses were downgraded by the full-semantic framework: 118 from structured Full to Partial and 7 from structured Partial to Discordant. Only 9/125 downgrades met the contradiction-only criterion (7 categorical standardization of highly selected upfront CRS and 2 bundling IPCT with CRS as standard treatment).

**Panel B.** Observed clinically consequential discordance categories

| **Category** | **Flagged responses, n** | **Independent scenarios** | **Observed pattern** |
| --- | --- | --- | --- |
| Incorrect treatment sequence | 9 | 3 (MET-C04, SYN-C08, SYN-C15) | Treatment selected at the wrong stage, including CRS despite high-risk timing, first-line progression, or signet-ring sequencing. |
| Categorical overstatement | 7 | 1 (GRY-01) | Highly selected upfront CRS described as standard, routine, or mandatory care. |
| Undertreatment | 5 | 1 (GRY-06) | Postoperative systemic therapy omitted after complete upfront CRS, with surveillance selected instead. |
| Overtreatment | 0 | 0 | No observed response met the prespecified overtreatment definition. |
| Failure to recognize unresectability | 0 | 0 | No observed response met the prespecified definition. |
| Inappropriate IPCT | 0 | 0 | No observed response met the prespecified definition. |

Twenty-one of 1,080 primary responses (1.9%) met a prespecified clinically consequential discordance flag, arising from 5/36 independent primary scenarios (13.9%; Wilson 95% CI 6.1%-28.7%). These are benchmark-level observations and should not be interpreted as independent patient-level safety events.

# Supplementary Reference

1. Schultz KS, Bansal VV, Wach MM, et al. Consensus Guideline for the Management of Colorectal Cancer with Peritoneal Metastases. Ann Surg Oncol. 2026;33:5260-5282. doi:10.1245/s10434-025-17363-0
